# Allogeneic stem cell-engineered EGFRvIII-specific CAR-NKT cells for treating glioblastoma with enhanced efficacy and safety

**DOI:** 10.1016/j.ymthe.2025.09.026

**Published:** 2025-09-12

**Authors:** Yan-Ruide Li, Yichen Zhu, Zhe Li, Xinyuan Shen, Tyler Halladay, Christopher Tse, Yanxin Tian, Jie Huang, Annabel S. Zhao, Nathan Y. Ma, Catherine Zhang, David A. Nathanson, Robert M. Prins, Lili Yang

**Affiliations:** 1 Department of Microbiology, Immunology & Molecular Genetics, University of California, Los Angeles, Los Angeles, CA 90095, USA; 2 Department of Bioengineering, University of California, Los Angeles, Los Angeles, CA 90095, USA; 3 Department of Molecular and Medical Pharmacology, University of California, Los Angeles, Los Angeles, CA 90095, USA; 4 Eli and Edythe Broad Center of Regenerative Medicine and Stem Cell Research, University of California, Los Angeles, Los Angeles, CA 90095, USA; 5 Jonsson Comprehensive Cancer Center, David Geffen School of Medicine, University of California, Los Angeles, Los Angeles, CA 90095, USA; 6 Molecular Biology Institute, University of California, Los Angeles, Los Angeles, CA 90095, USA; 7 Parker Institute for Cancer Immunotherapy, University of California, Los Angeles, Los Angeles, CA 90095, USA; 8 Goodman-Luskin Microbiome Center, University of California, Los Angeles, Los Angeles, CA 90095, USA

**Keywords:** glioblastoma, GBM, allogeneic CAR-NKT cells, EGFRvIII-targeting CAR, allogeneic cell therapy, off-the-shelf, orthotopic model, GBM patient-derived xenograft mouse model, high safety profile, cytokine release syndrome

## Abstract

Glioblastoma (GBM) is the most aggressive and lethal primary brain tumor in adults, characterized by resistance to standard therapies, including surgical resection, radiation, chemotherapy, and targeted agents. While chimeric antigen receptor (CAR)-engineered T (CAR-T) cell therapy has emerged as a promising immunotherapeutic approach for GBM, its application remains limited by tumor antigen escape, an immunosuppressive tumor microenvironment (TME), treatment-associated toxicities such as cytokine release syndrome (CRS), and the logistical complexities of autologous cell manufacturing. In this study, we leveraged hematopoietic stem and progenitor cell (HSPC) gene engineering combined with a feeder-free, *ex vivo* differentiation protocol to generate allogeneic EGFRvIII-specific CAR-engineered invariant natural killer T (^Allo^ECAR-NKT) cells through a clinically guided, scalable platform. These cells exhibit potent, multifaceted antitumor activity against GBM, including direct tumor cell killing via CAR and NK receptors and selective targeting of CD1d^+^ immunosuppressive cells within the TME via their invariant T cell receptors. In both subcutaneous and orthotopic GBM humanized models, ^Allo^ECAR-NKT cells demonstrated robust efficacy, minimal systemic leakage from the brain, and a reduced risk of CRS. Collectively, our findings support ^Allo^ECAR-NKT cells as a next-generation, off-the-shelf immunotherapy with enhanced efficacy and safety for the treatment of GBM.

## Graphical abstract

**Figure:**
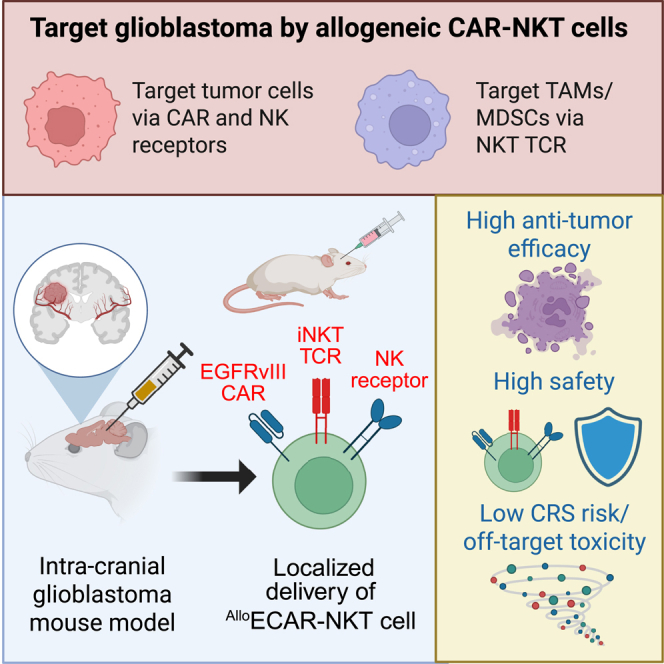

## Teaser

Yang and colleagues developed allogeneic CAR-NKT cells targeting EGFRvIII for glioblastoma. These cells outperformed conventional CAR-T cells in preclinical mouse models, showing enhanced antitumor activity and improved safety, including limited systemic distribution and reduced risk of cytokine release syndrome, highlighting a clinically translatable off-the-shelf immunotherapy for glioblastoma.

## Introduction

Glioblastoma (GBM), classified by the World Health Organization as a grade IV adult-type isocitrate dehydrogenase-wild-type astrocytoma, is the most aggressive and lethal primary brain tumor in adults.^1^^,^^2^ It predominantly originates in the frontal and temporal lobes of the supratentorial cerebral hemispheres.^1^ GBM is highly resistant to conventional therapies, including surgical resection, radiotherapy, chemotherapy, and targeted agents. Despite advancements in these modalities, the average overall survival remains limited to 12–15 months, and recurrence is evidently inevitable.^1^^,^^3^ Long-term survival is rare, with fewer than 5.8% of patients surviving beyond 5 years. Among patients with recurrent GBM, only ∼15% achieve a progression-free survival of approximately 6 months.^1^^,^^3^ These clinical outcomes highlight the urgent need for more effective and innovative therapeutic strategies.

Immunotherapy has emerged as a promising approach for GBM, driven by its success in other malignancies.^4^ Ongoing pre-clinical and clinical investigations are exploring several immunotherapeutic modalities, including immune checkpoint inhibitors, cancer vaccines, oncolytic viruses, and adoptive T cell therapies.^4^ Among these therapies, chimeric antigen receptor (CAR)-engineered T (CAR-T) cells delivered locoregionally have demonstrated a favorable safety profile and transient antitumor activity in multiple early-phase clinical trials for GBM (NCT02209376, NCT05168423, and NCT02208362), thus highlighting their potential.^5^^,^^6^

CAR-T cell therapy targeting epidermal growth factor receptor variant III (EGFRvIII) has emerged as a novel and tumor-specific immunotherapeutic strategy for GBM.^3^^,^^7^^,^^8^^,^^9^ EGFR is a transmembrane receptor tyrosine kinase altered in approximately 60% of GBM tumors through mutation, amplification, rearrangement, and/or alternative splicing.^3^ These aberrations promote oncogenesis by activating downstream signaling pathways such as PI3K/AKT/mTOR and Ras/Raf/MEK/ERK, which facilitate tumor growth and survival.^3^ EGFRvIII, one of the most prevalent EGFR variants in GBM, is defined by deletions of exons 2–7, which induce constitutive activation of the receptor independent of ligand binding.^3^^,^^4^ This mutant is expressed in approximately 30% of GBM tumors and is absent in normal tissues, making it an attractive and selective target for CAR-T cell therapy.^3^ Several clinical trials investigating EGFRvIII-specific CAR-T (ECAR-T) cells are under way to evaluate their safety and efficacy in GBM.^7^^,^^8^

Despite their therapeutic potential, conventional ECAR-T cells face several critical limitations, and clinical outcomes have remained modest to date.^4^^,^^10^ A major barrier is the highly immunosuppressive tumor microenvironment (TME) characteristic of GBM, which impairs CAR-T cell infiltration, persistence, and function.^4^^,^^10^^,^^11^^,^^12^^,^^13^ Additionally, tumor antigen heterogeneity and antigen downregulation facilitate immune evasion and limit sustained therapeutic responses.^10^^,^^11^ Moreover, current ECAR-T cell therapies are autologous and require patient-specific cell manufacturing. This individualized process is financially costly, time-intensive, and difficult to scale, thereby limiting broader patient access and hindering the implementation of combination therapies, an approach that may be necessary for improving outcomes in GBM.^4^^,^^10^^,^^14^ To overcome these challenges and fully harness the therapeutic potential of CAR-directed therapies, the development of potent off-the-shelf alternatives capable of addressing these challenges is imperative.

To address these limitations, we leveraged our previously established hematopoietic stem and progenitor cell (HSPC) gene engineering platform in combination with a clinically guided, *ex vivo* feeder-free differentiation culture system to generate allogeneic EGFRvIII-specific CAR-engineered natural killer T (^Allo^ECAR-NKT) cells with high yield and purity.^15^ These cells integrate features of both NK and T cells, allowing tumor recognition through both CAR-dependent mechanisms and innate NK receptor pathways. Additionally, ^Allo^ECAR-NKT cells can target immunosuppressive TME cell populations, including myeloid-derived suppressor cells (MDSCs) and tumor-associated macrophages/microglia (TAMs), via their invariant NKT T cell receptor (TCR) in a CD1d-restricted manner. Utilizing a comprehensive suite of preclinical models, including primary GBM patient-derived neurospheres, established GBM tumor cell lines, *in vitro* cytotoxicity assays, and *in vivo* subcutaneous (s.c.) and orthotopic xenograft models, we demonstrate that ^Allo^ECAR-NKT cells exert robust, multifaceted antitumor activity against GBM, while exhibiting a favorable safety profile. These findings support the translational potential of ^Allo^ECAR-NKT cells as a next-generation, off-the-shelf immunotherapy for GBM.

## Results

### Generate HSPC-derived allogeneic stem cell-engineered EGFRvIII-specific CAR-NKT cells using a clinically guided culture method

**Figure 1:**
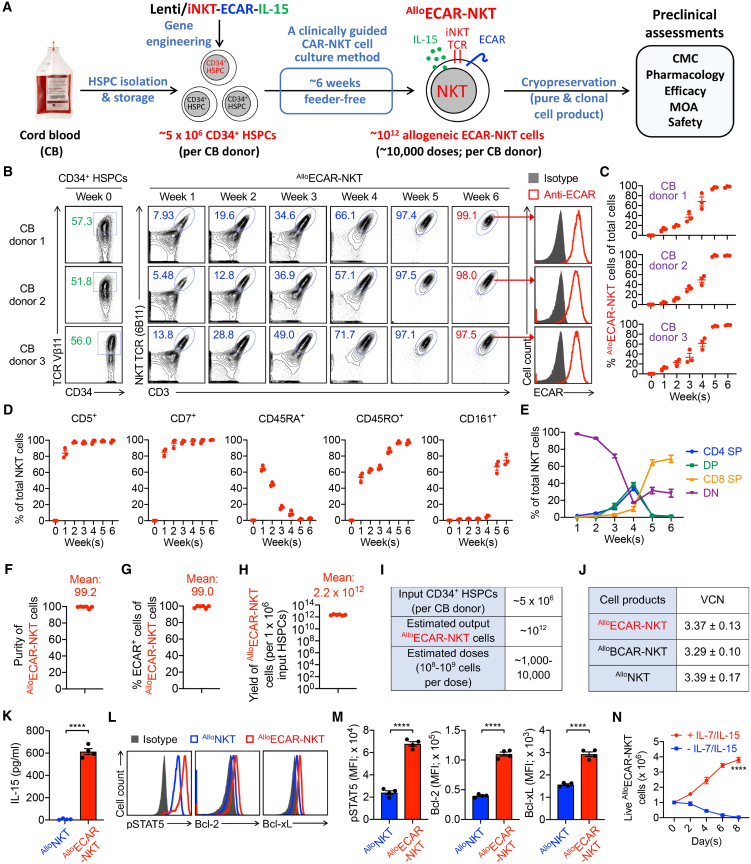
HSPC-derived allogeneic ECAR-NKT cells can be generated using a clinically guided culture method with high yield, purity, and robustness (A) Schematics showing the generation of ^Allo^ECAR-NKT cells. CMC, chemistry, manufacturing, and controls; HSPC, hematopoietic stem and progenitor cells; Lenti/iNKT-ECAR-IL-15, lentiviral vector encoding a pair of iNKT TCR α and β chains, an EGFRvIII-specific CAR, and a human soluble IL-15; MOA, mechanism of action. (B) FACS monitoring of the generation of ^Allo^ECAR-NKT cells during the 6-week culture. Intracellular iNKT TCR was stained using a TCR Vβ11 monoclonal antibody, surface iNKT TCR was stained using a 6B11 monoclonal antibody, and ECAR was stained using an F(ab′)2 antibody. Data generated from 3 different CB donors are shown. (C) Percentage of ^Allo^ECAR-NKT cells in total live cells during the 6-week culture (*n* = 3; *n* indicates different culture batches). (D) Percentage of ^Allo^ECAR-NKT cells expressing the indicated markers (i.e., CD5, CD7, CD45RA, CD45RO, and CD161) among total ^Allo^ECAR-NKT cells during the 6-week culture (*n* = 3; *n* indicates different CB donors). (E) Percentage of the subpopulations of ^Allo^ECAR-NKT cells during the 6-week culture (*n* = 3; *n* indicates different CB donors). (F–H) The purity (F), ECAR^+^ percentage (G), and yield (H) of ^Allo^ECAR-NKT cells (*n* = 6). (I) Table showing the estimated output cell numbers and doses of ^Allo^ECAR-NKT cells generated from 1 CD donor. (J) Table showing the vector copy number (VCN) of ^Allo^ECAR-NKT cells. The allogeneic IL-15-enhanced BCMA-specific CAR-NKT (^Allo^BCAR-NKT) and allogeneic HSPC-engineered NKT (^Allo^NKT) were included as controls. Note that ^Allo^NKT cells were not engineered with IL-15 transgene. (K) ELISA analyses of IL-15 production by ^Allo^ECAR-NKT cells (*n* = 4). (L and M) FACS detection (L) and quantification (M) of IL-15-related biomarker expression in ^Allo^ECAR-NKT and ^Allo^NKT cells (*n* = 4). (N) *In vitro* dysregulated growth assay. ^Allo^ECAR-NKT cells were cultured *in vitro* with/without addition of IL-7/IL-15, followed by quantification of live cells over time (*n* = 4). Representative of >6 experiments. Data are presented as the mean ± SEM. ∗∗∗∗*p* < 0.0001, by Student’s t test (K, M, and N).

We have previously established a robust technology platform for generating allogeneic CAR-NKT cells using lentiviral engineering of HSPCs followed by a clinically guided culture method.^15^ Using this platform, we successfully developed BCMA-specific and CD33-specific CAR-NKT cells to target multiple myeloma and acute myeloid leukemia.^15^^,^^16^ Building on this foundation, we applied our approach to develop allogeneic CAR-NKT cells targeting solid tumors, with a specific focus on GBM (Figure 1A). EGFRvIII is a tumor-specific mutation highly expressed in malignant GBM and minimally present in normal tissues, making it an attractive and selective immunotherapeutic target.^17^^,^^18^ Accordingly, we engineered ^Allo^ECAR-NKT cells from human HSPCs to target EGFRvIII^+^ GBM (Figure 1A).

CD34^+^ HSPCs were obtained from human umbilical cord blood (CB) through commercial sources such as HemaCare. These HSPCs were transduced with a lentiviral vector encoding 3 key components: (1) a pair of invariant NKT TCR α and β chains derived from healthy donor peripheral blood mononuclear cells (PBMCs), (2) an EGFRvIII-specific CAR, and (3) a soluble human interleukin-15 (IL-15) transgene (Figures S1A and S1B). The invariant NKT (iNKT) TCR construct has been previously validated for its ability to reprogram HSPCs and direct their differentiation into functional NKT cells.^19^^,^^20^ The ECAR was constructed using a single-chain variable fragment derived from a monoclonal antibody specific for EGFRvIII, enabling selective recognition and targeting of EGFRvIII-expressing tumor cells.^7^ Soluble IL-15 was included to enhance the *in vivo* persistence of CAR-NKT cells, as demonstrated in both HSPC- and PBMC-derived CAR-NKT cells.^15^^,^^16^^,^^21^^,^^22^^,^^23^ The lentiviral vector achieved consistent and efficient gene delivery, with transduction rates exceeding 50% across all tested CB HSPC batches, thereby ensuring reliable downstream generation of ^Allo^ECAR-NKT cells (Figure 1B).

The transduced HSPCs were cultured *ex vivo* using a scalable, clinically guided 6-week protocol to generate ^Allo^ECAR-NKT cells. The protocol comprises 4 stages: stage 1 HSPC expansion (∼2 weeks), stage 2 NKT differentiation (∼1 week), stage 3 NKT deep differentiation (∼1 week), and stage 4 NKT expansion (∼2 weeks).^15^^,^^24^ Throughout this process, HSPCs progressively differentiated into NKT cells (Figure 1B). While differentiation efficiency varied slightly across CB donors and batches, the final cell product consistently exhibited high purity, with >97% of cells identified as NKT TCR^+^CD3^+^ and minimal contamination by endogenous αβ T cells (NKT TCR^−^CD3^+^) (Figures 1B and 1C). This high purity is critical for ensuring product safety, minimizing the risk of graft-versus-host disease (GvHD) associated with residual conventional T cells.^25^^,^^26^ Importantly, the NKT TCR and ECAR transgenes were co-expressed from the same lentiviral construct, enabling TCR-mediated positive selection during NKT cell differentiation and resulting in uniform CAR expression across the ^Allo^ECAR-NKT cell population (Figure 1B).^27^^,^^28^^,^^29^ Over 99% of the differentiated NKT cells expressed ECAR, eliminating the need for further CAR^+^ cell enrichment. This streamlined approach yields a clonal, highly pure ^Allo^ECAR-NKT cell product suitable for downstream therapeutic applications.

The development of ^Allo^ECAR-NKT cells recapitulated the canonical trajectory of NKT cell maturation.^30^^,^^31^ Upon differentiation, the cells robustly expressed pan-T cell markers such as CD5 and CD7, with sustained high expression throughout the culture period (Figure 1D). As differentiation progressed, the cells exhibited a phenotypic transition from a naive to a memory state, characterized by upregulation of CD45RO and downregulation of CD45RA (Figure 1D). During the expansion phase, mature ^Allo^ECAR-NKT cells further acquired expression of the NK-associated receptor CD161, consistent with a cytotoxic effector phenotype (Figure 1D).

The expression dynamics of CD4 and CD8 co-receptors followed a well-defined developmental pathway.^30^^,^^31^ The differentiating cells initially displayed a double-negative (DN) (CD4^−^CD8^−^) phenotype, transitioned through a double-positive (CD4^+^CD8^+^) stage, and ultimately matured into either DN or CD8 single-positive (CD8 SP) subsets (Figure 1E). Notably, the final ^Allo^ECAR-NKT cell product was predominantly composed of DN and CD8 SP populations, with minimal representation of CD4^+^ cells, which is an outcome consistent with other *in vitro* NKT differentiation protocols (Figure 1E).^19^^,^^32^^,^^33^^,^^34^ Given that DN and CD8 SP NKT cells possess potent cytotoxic activity, their enrichment in the final product is advantageous for cancer immunotherapy applications.^35^^,^^36^^,^^37^

In addition to their high purity and consistent CAR expression, ^Allo^ECAR-NKT cells exhibited robust yield (Figures 1F–1H). From a single CB donor containing ∼5 × 10^6^ CD34^+^ HSPCs, an estimated 10^12^ mature ^Allo^ECAR-NKT cells could be generated (Figures 1H and 1I). Given that current autologous CAR-T cell therapies typically require 10^8^–10^9^ cells per treatment, a single manufacturing run could potentially yield 1,000–10,000 therapeutic doses, highlighting the scalability of this platform (Figure 1I). Importantly, the incorporation of different CAR constructs, or the absence thereof, did not significantly impact the overall yield of HSPC-derived NKT cells, underscoring the robustness and versatility of the manufacturing process (Figures 1I and S1).^15^^,^^16^ Furthermore, the vector copy number (VCN) in the resulting ^Allo^ECAR-NKT cells was approximately 3 copies per genome, well within the accepted safety threshold of ≤5 copies for clinical-grade lentiviral or retroviral CAR-T cell products (Figure 1J).^38^ This observation supports the potential safety of ^Allo^ECAR-NKT cells with regard to the risk of insertional mutagenesis.

We further evaluated the impact of the IL-15 transgene in the ^Allo^ECAR-NKT cell products. Compared to allogeneic HSPC-derived NKT (^Allo^NKT) cells lacking CAR and IL-15 engineering (Figure S1), the IL-15-engineered ^Allo^ECAR-NKT cells secreted significantly higher levels of IL-15 and exhibited elevated expression of IL-15 signaling-associated biomarkers, including phosphorylated STAT5 (pSTAT5), Bcl-2, and Bcl-xL (Figures 1K–1M).^39^^,^^40^ To assess the potential for dysregulated proliferation, we performed an *in vitro* dysregulated growth assay. ^Allo^ECAR-NKT cells failed to survive in the absence of exogenous IL-7 and IL-15, indicating that these cells retain cytokine dependence and suggesting a low risk of dysregulated or uncontrolled cell growth (Figure 1N).

In addition, we evaluated the expansion of ^Allo^ECAR-NKT cells under different cytokine conditions, including IL-7 alone and the combination of IL-15 and IL-7 (Figure S2A). We observed that culturing with IL-7 alone significantly reduced the expansion efficiency of ^Allo^ECAR-NKT cells, indicating that endogenous IL-15 production by these cells is insufficient to support robust proliferation (Figure S2B). These findings suggest that exogenous supplementation with both IL-15 and IL-7 is essential during the expansion phase to ensure optimal cell maturation and yield, despite the intrinsic IL-15 secretion by ^Allo^ECAR-NKT cells.

In conclusion, we successfully generated HSPC-engineered ^Allo^ECAR-NKT cells using a clinically guided, scalable culture method. The process yielded high-purity, clonally engineered NKT cells with robust CAR expression and consistent production across donors. We comprehensively evaluated their chemistry, manufacturing, and controls, including yield, phenotype, VCN, and safety profile. These ^Allo^ECAR-NKT cells represent a promising off-the-shelf cellular immunotherapy candidate for GBM, and we subsequently evaluated their phenotype, functionality, antitumor efficacy, and safety.

### Allogeneic ECAR-NKT cells resemble endogenous human NKT cells with strong effector and cytotoxic functions

**Figure 2:**
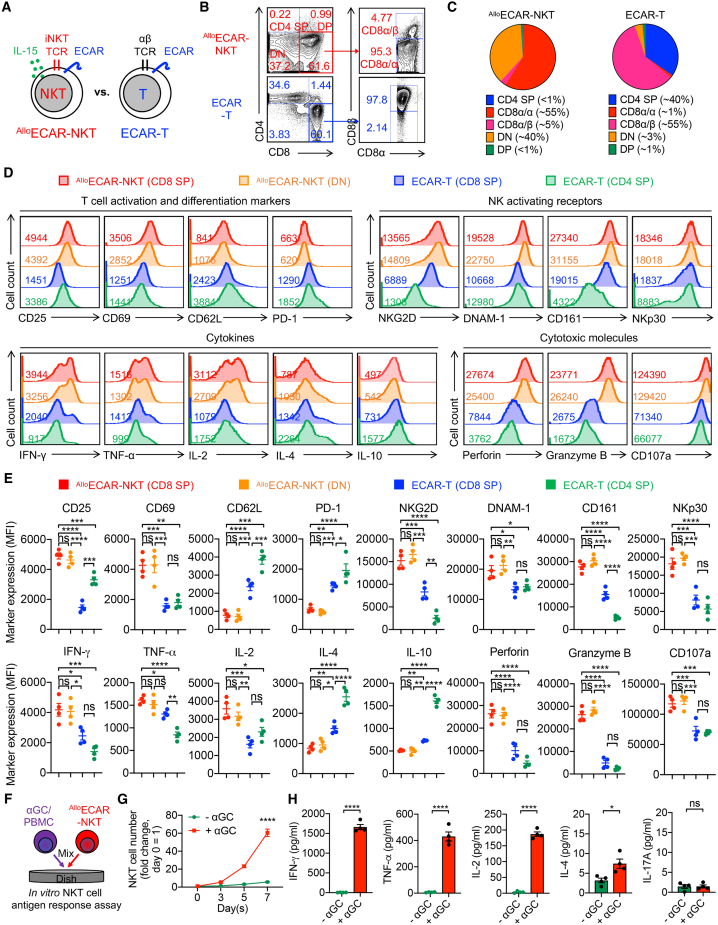
Allogeneic ECAR-NKT cells display typical NKT cell characteristics with strong effector and cytotoxic functions (A) Experimental design to compare the phenotype and functionality between ^Allo^ECAR-NKT cells and healthy donor PBMC-derived ECAR-engineered conventional T (ECAR-T) cells. (B) FACS detection of CD4/CD8 expression on the indicated cells. (C) Comparison of the indicated subpopulation percentages between ^Allo^ECAR-NKT and conventional ECAR-T cells. (D) FACS detection of surface and intracellular marker expression in the indicated cell subpopulations. (E) Quantification of (D) (*n* = 4). (F–H) Antigen responses of ^Allo^ECAR-NKT cells. ^Allo^ECAR-NKT cells were stimulated with/without α-galactosylceramide (αGC)-loaded PBMCs for 1 week. (F) Experimental design. (G) Growth curve of ^Allo^ECAR-NKT cells (*n* = 4). (H) ELISA measurements of cytokine (IFN-γ, TNF-α, IL-2, IL-4, and IL-17A) levels in the culture supernatants collected on day 7 (*n* = 4). Representative of 3 experiments. Data are presented as the mean ± SEM. ns, not significant, ∗*p* < 0.05, ∗∗*p* < 0.01, ∗∗∗*p* < 0.001, ∗∗∗∗*p* < 0.0001, by Student’s t test (G and H) or 1-way ANOVA (E).

To further characterize the phenotype and functionality of ^Allo^ECAR-NKT cells, we performed a side-by-side comparison with healthy donor PBMC-derived conventional ECAR-T cells (Figures 2A and S3A–S3E). While ECAR-T cells exhibited high (>60%) CAR expression, not all cells expressed the CAR, in contrast to ^Allo^ECAR-NKT cells, in which nearly 100% of the population co-expressed ECAR (Figures S3C and S3D). Importantly, neither ^Allo^ECAR-NKT nor ECAR-T cells expressed EGFRvIII themselves, suggesting that these effector cells would not be subject to self-targeting (Figure S3F).

We first analyzed the CD4/CD8 subpopulations of ^Allo^ECAR-NKT and conventional ECAR-T cells. ^Allo^ECAR-NKT cells predominantly consisted of CD8 SP and DN populations (Figure 1E). Within the CD8 SP subset, the majority expressed the CD8α/α homodimer, with a smaller fraction expressing the CD8α/β heterodimer (Figures 2B and 2C). Functionally, both CD8α/α and DN human NKT cells exhibited similar profiles, characterized by pro-inflammatory cytokine production and potent cytotoxic activity.^41^^,^^42^ In contrast, conventional ECAR-T cells comprised both CD4 SP and CD8 SP populations, with the CD8 SP subset primarily expressing the CD8α/β heterodimer and minimal CD8α/α expression (Figures 2B, 2C, and S3G). These subpopulation differences suggest that ^Allo^ECAR-NKT cells are enriched for cytotoxic CD8α/α and DN subsets, whereas ECAR-T cells contain a mixture of CD4 SP and CD8α/β SP cells (Figure 2C). To further define the phenotypic and functional differences among these populations, we performed a comparative analysis of T and NK cell surface markers, intracellular cytokines, and cytotoxic molecules using flow cytometry.

We first found that the CD8α/α and DN subsets within ^Allo^ECAR-NKT cells displayed highly similar phenotypes, with comparable expression of all markers analyzed (Figures 2D and 2E). This observation aligns with prior reports of endogenous human NKT cells, where CD8α/α and DN cells share similar functional characteristics.^41^^,^^42^ ^Allo^ECAR-NKT cells exhibited significantly higher expression of T cell activation markers, including CD25 and CD69, compared to CD4 SP and CD8 SP ECAR-T cells, with CD8 SP ECAR-T cells showing the lowest expression (Figures 2D and 2E). Conversely, ^Allo^ECAR-NKT cells expressed the lowest levels of CD62L, suggesting a shift toward an effector or effector memory phenotype, in contrast to the more central memory-like profile of conventional ECAR-T cells (Figures 2D and 2E).^43^ Moreover, ^Allo^ECAR-NKT cells had reduced PD-1 expression, indicating a lower exhaustion profile compared to conventional ECAR-T cells (Figures 2D and 2E).

Importantly, ^Allo^ECAR-NKT cells expressed markedly higher levels of activating NK receptors (NKRs), including NKG2D, DNAM-1, CD161, and NKp30, compared to both CD8 SP and CD4 SP ECAR-T cells (Figures 2D and 2E). This highlights their innate-like cytotoxic potential and suggests NKR-mediated tumor targeting as a key functional mechanism.^33^^,^^44^^,^^45^ In terms of cytokine production, ^Allo^ECAR-NKT cells expressed significantly higher levels of T helper 1 cell (Th1)-associated cytokines (interferon-γ [IFN-γ], tumor necrosis factor α [TNF-α], and IL-2) and lower levels of Th2-like cytokines (IL-4 and IL-10), consistent with their cytotoxic CD8 SP and DN phenotypes (Figures 2D and 2E). Additionally, ^Allo^ECAR-NKT cells produced elevated levels of cytotoxic effector molecules, including perforin, granzyme B, and CD107a, relative to conventional ECAR-T cells (Figures 2D and 2E). These data collectively demonstrate the highly activated, minimally exhausted, and intrinsically cytotoxic phenotype of ^Allo^ECAR-NKT cells, underscoring their potential as potent tumor cell killers.

In addition to comparisons with conventional ECAR-T cells, we conducted a side-by-side phenotypic analysis of ^Allo^ECAR-NKT cells and PBMC-derived ECAR-NKT (^PBMC^ECAR-NKT) cells, which were generated by sorting endogenous NKT cells from healthy donors, followed by α-galactosylceramide (α-GalCer or αGC; an NKT agonist glycolipid antigen) stimulation and lentiviral transduction (Figure S3H).^46^ We routinely achieved high-purity ^PBMC^ECAR-NKT cells with robust CAR expression (Figure S3I). ^PBMC^ECAR-NKT cells exhibited a heterogeneous CD4/CD8 co-receptor profile, consisting of CD4 SP, CD8 SP, and DN populations. Among the CD8 SP subset, most cells expressed the CD8α/α isoform, with fewer expressing CD8α/β (Figures S3I and S3J). This diversity contrasts with the more uniform CD4/CD8 profile observed in ^Allo^ECAR-NKT cells (Figures 2B and 2C). Phenotypically, both ^Allo^ECAR-NKT cells and ^PBMC^ECAR-NKT cells exhibited high expression of CD161 and CD69, low expression of CD62L, and elevated levels of cytotoxic molecules, including perforin and granzyme B (Figures S3K and S3L). Compared to ^PBMC^ECAR-NKT cells, ^Allo^ECAR-NKT cells expressed significantly higher levels of NKRs, including NKG2D, DNAM-1, and NKp46 (Figures S3K and S3L). ^Allo^ECAR-NKT cells also produced increased amounts of Th1-type cytokines (e.g., IFN-γ, TNF-α) but reduced levels of Th2-type cytokines (e.g., IL-4, IL-10) (Figures S3K and S3L). These results suggest that while ^Allo^ECAR-NKT cells phenotypically resemble ^PBMC^ECAR-NKT cells, they exhibit enhanced cytotoxic and Th1-skewed immune profiles, making them suitable for potent cancer immunotherapy applications.

To evaluate the functionality of the introduced NKT TCR, ^Allo^ECAR-NKT cells were stimulated with the glycolipid antigen αGC (Figure 2F). Upon stimulation, these cells demonstrated robust proliferative responses and secreted high levels of Th1-associated cytokines, including IFN-γ, TNF-α, and IL-2 (Figures 2G and 2H). In contrast, they produced relatively low levels of Th2- and Th17-associated cytokines, such as IL-4 and IL-17A (Figures 2G and 2H). These findings indicate a Th1-skewed functional profile of ^Allo^ECAR-NKT cells, which is consistent with their predominant CD8 SP and DN phenotypes.

### Allogeneic ECAR-NKT cells kill GBM tumor cells at high efficacy and use multiple targeting mechanisms

^Allo^ECAR-NKT cells are expected to target GBM tumor cells through multiple mechanisms, including direct killing of EGFRvIII-expressing tumor cells via ECAR-mediated antigen targeting, and recognition of GBM tumor cells through activating NKRs engaging NK ligands.^35^^,^^47^^,^^48^ Thus, the multi-targeting capability of ^Allo^ECAR-NKT cells enables them to overcome immune evasion by addressing tumor antigen heterogeneity and limiting antigen escape, unlike conventional CAR-T cells that are restricted to a single target.^10^

**Figure 3:**
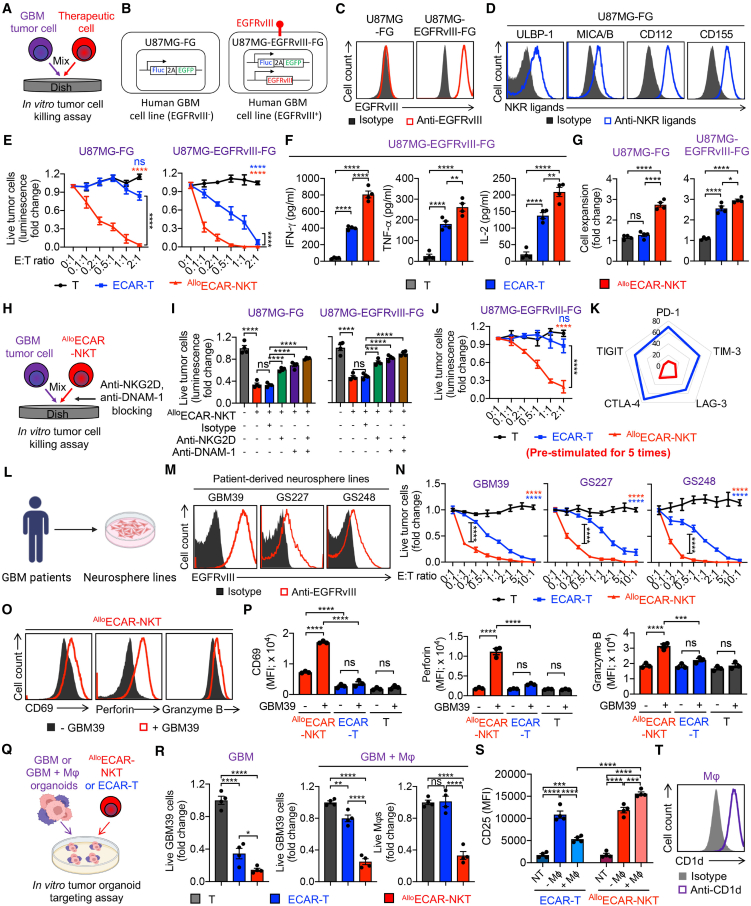
Allogeneic ECAR-NKT cells demonstrate potent *in vitro* antitumor efficacy (A–G) Study of the *in vitro* antitumor efficacy of ^Allo^ECAR-NKT cells against human GBM cell lines. ECAR-T cells and non-ECAR-engineered T cells were included as therapeutic cell controls. (A) Experimental design. (B) Schematics showing the indicated human GBM cell lines. U87MG-FG, U87MG cell line engineered to overexpress the firefly luciferase and green fluorescent protein dual reporters (FG); U87MG-EGFRvIII-FG, U87MG-FG cell line engineered to overexpress EGFRvIII. (C) FACS detection of EGFRvIII expression on the indicated GBM cells. (D) FACS detection of NKR ligand expression on the U87MG-FG tumor cells. (E) Tumor cell killing data at 24 h (*n* = 4). (F) ELISA analyses of production of proinflammatory cytokines by the indicated therapeutic cells (*n* = 4). (G) Cell counts of the indicated therapeutic cells following co-culture with tumor cells (*n* = 4). (H and I) Study of the tumor cell killing mechanisms of ^Allo^ECAR-NKT cells mediated by NKRs (i.e., NKG2D and DNAM-1). (H) Experimental design. (I) Tumor cell killing data at 24 h (E:T ratio = 0.2:1 for U87MG-FG, E:T ratio = 0.1:1 for U87MG-EGFRvIII-FG; *n* = 4). (J and K) Study of the long-term *in vitro* antitumor efficacy of ^Allo^ECAR-NKT cells. Therapeutic cells were subjected to 5 rounds of stimulation with tumor cells, followed by collection and subsequent analysis. (J) Tumor cell killing data (*n* = 4). (K) Radar plots showing the immune checkpoint expression in the indicated therapeutic cells (*n* = 4). (L–P) Studying the *in vitro* antitumor efficacy of ^Allo^ECAR-NKT cells against primary GBM patient-derived neurosphere lines. Three cell lines with varying levels of EGFRvIII expression were included. (L) Experimental design. (M) FACS detection of EGFRvIII expression on the indicated neurosphere lines. (N) Tumor cell killing data at 24 h (*n* = 4) (O) FACS detection of surface activation marker (i.e., CD69) and intracellular cytotoxic molecules (i.e., perforin and granzyme B) of ^Allo^ECAR-NKT cells 24 h after co-culture with GBM39 cells. (P) Quantification of (O) (*n* = 3). (Q–T) Study of ^Allo^ECAR-NKT cells targeting of GBM TME using GBM/macrophage co-culture organoid models. (Q) Experimental design. (R) Tumor cell and macrophage killing data at 24 h (*n* = 4) (S) FACS analyses of activation marker (i.e., CD25) expression in the indicated therapeutic cells. (T) FACS detection of CD1d expression on macrophages. Representative of 3 experiments. Data are presented as the mean ± SEM. ns, not significant, ∗*p* < 0.05, ∗∗*p* < 0.01, ∗∗∗*p* < 0.001, ∗∗∗∗*p* < 0.0001, by 1-way ANOVA (F, G, I, P, R, and S) or 2-way ANOVA (E, J, and N).

The CAR- and NKR-mediated targeting mechanisms of ^Allo^ECAR-NKT cells were validated through a series of *in vitro* tumor cell killing assays using 2 human GBM cell lines: U87MG (EGFRvIII^−^) and U87MG-EGFRvIII (engineered to overexpress EGFRvIII) (Figures 3A–3C). Both cell lines expressed high levels of NKR ligands, including ULBP and MICA/B (ligands for NKG2D), as well as CD112 and CD155 (ligands for DNAM-1) (Figure 3D). To enable real-time monitoring of tumor cell viability, both GBM lines were further engineered to express firefly luciferase and green fluorescent protein (GFP) dual reporters (FG), allowing assessment via luciferase-based cytotoxicity assays and flow cytometry (Figure 3B). The antitumor activities of ^Allo^ECAR-NKT cells were compared with conventional ECAR-T cells and non-engineered PBMC-derived T cells across these assays.

Unmodified T cells failed to kill either GBM tumor cell line within a 24-h period (Figure 3E). However, when engineered with ECAR, conventional ECAR-T cells exhibited robust cytotoxicity against U87MG-EGFRvIII-FG cells, while showing no killing activity toward EGFRvIII^−^ tumor cells, confirming their strict dependence on CAR-antigen recognition (Figure 3E). In contrast, ^Allo^ECAR-NKT cells demonstrated potent cytotoxicity against both EGFRvIII^+^ and EGFRvIII^−^ tumor cells, with enhanced killing observed against EGFRvIII^+^ cells (Figure 3E). This indicates that ^Allo^ECAR-NKT cells mediate tumor cell killing through both CAR-dependent and CAR-independent mechanisms. Notably, the tumor-killing capacity of the therapeutic cells correlated with their proliferation and secretion of pro-inflammatory cytokines, including IFN-γ, TNF-α, and IL-2, with ^Allo^ECAR-NKT cells exhibiting the strongest expansion and cytokine production (Figures 3F and 3G). Importantly, ^Allo^ECAR-NKT cells effectively eliminated EGFRvIII^−^ tumor cells, and this cytotoxic activity was significantly reduced upon blockade of NKR pathways (i.e., NKG2D and DNAM-1), highlighting the critical role of NKR-mediated tumor recognition and killing (Figures 3H and 3I).

Furthermore, we assessed the long-term tumor-killing capacity of ^Allo^ECAR-NKT cells through repeated tumor challenge assays. After 5 rounds of tumor exposure, ^Allo^ECAR-NKT cells consistently maintained significantly higher levels of cytotoxicity compared to conventional ECAR-T cells (Figures 3J, S4A, and S4B). This sustained functionality is likely attributed to their lower expression of exhaustion-associated markers, including PD-1, CTLA-4, TIM-3, LAG-3, and TIGIT (Figure 3K). These features contribute to the enhanced persistence and prolonged effector function of ^Allo^ECAR-NKT cells *in vivo*, supporting their superior antitumor efficacy over time.

We then utilized patient-derived GBM neurosphere lines to further validate the antitumor capacity of ^Allo^ECAR-NKT cells (Figure 3L; Table S1). These neurospheres exhibited heterogeneous expression of the CAR target antigen EGFRvIII (Figure 3M). *In vitro* tumor killing assays demonstrated that ^Allo^ECAR-NKT cells exhibited the highest cytotoxic efficacy against these neurosphere lines compared to conventional T cells and ECAR-T cells (Figure 3N). This superior killing correlated with elevated expression of activation markers (i.e., CD69) and increased production of cytotoxic molecules (i.e., perforin and granzyme B) (Figures 3O and 3P). In conclusion, ^Allo^ECAR-NKT cells show robust antitumor activity through multiple targeting mechanisms against diverse GBM tumor cells, including both CAR antigen-positive and antigen-negative populations. This suggests their potential to overcome CAR antigen escape, a common limitation of conventional CAR-T cell therapies.^10^

### Allogeneic ECAR-NKT cells target the immunosuppressive GBM TME via CD1d recognition

The immunosuppressive GBM TME represents a major barrier to the success of immunotherapies, including immune checkpoint inhibitors and CAR-T cells.^11^^,^^12^ GBM tumors actively recruit and expand immunosuppressive myeloid populations, such as MDSCs and TAMs, which contribute to tumor progression and therapy resistance.^11^^,^^12^ Targeting these immunosuppressive cells is therefore critical to achieving optimal therapeutic outcomes. Notably, both MDSCs and TAMs express high levels of CD1d, the ligand recognized by the NKT TCR, rendering them susceptible to NKT cell-mediated killing.^49^^,^^50^ We thus investigated the ability of ^Allo^ECAR-NKT cells to target and eliminate immunosuppressive cells within the GBM TME using an *in vitro* tumor organoid-targeting assay (Figure 3Q).

To model the immunosuppressive GBM microenvironment, we established GBM organoids by 3-dimensionally co-culturing U87MG-EGFRvIII tumor cells with M2-polarized macrophages derived from human monocytes to mimic TAMs (termed GBM/TAM organoids).^51^^,^^52^ *In vitro* cytotoxicity assays demonstrated that ^Allo^ECAR-NKT cells could effectively kill CD1d^+^ macrophages, while conventional ECAR-T cells lacked this capacity (Figures S4C and S4D). The cytotoxicity of ^Allo^ECAR-NKT cells was further enhanced by the addition of the NKT agonist αGC and attenuated upon CD1d blockade, confirming that the killing was mediated through the NKT TCR-CD1d axis (Figures S4C and S4D). Notably, ^Allo^ECAR-NKT cells did not exhibit cytotoxicity against the CD1d^−^ immune cells such as T, B, or NK cells (Figures S4E–S4G), supporting their safety profile and demonstrating their ability to spare normal immune cells while preserving host immune function.

We then evaluated the tumor cell killing capacity of ^Allo^ECAR-NKT cells within GBM/TAM organoids (Figure 3Q). In the absence of TAMs, both ECAR-T and ^Allo^ECAR-NKT cells effectively eliminated tumor cells, with ^Allo^ECAR-NKT cells exhibiting superior cytotoxicity (Figure 3R). However, in the presence of TAMs, ECAR-T cell function was markedly suppressed, indicating TAM-mediated immunosuppression (Figures 3R and 3S). In contrast, ^Allo^ECAR-NKT cells retained robust tumor-killing activity despite the immunosuppressive environment (Figures 3R and 3S). These findings suggest that ^Allo^ECAR-NKT cells can overcome TAM-mediated suppression and eliminate CD1d^+^ TAMs, thereby preserving their effector functions (Figure 3T). Collectively, these results highlight the dual-targeting potential of ^Allo^ECAR-NKT cells to simultaneously eliminate GBM tumor cells and remodel the TME, reducing immune resistance and enhancing therapeutic efficacy.

### Allogeneic ECAR-NKT cells exhibit superior antitumor efficacy in human GBM xenograft mouse models

**Figure 4:**
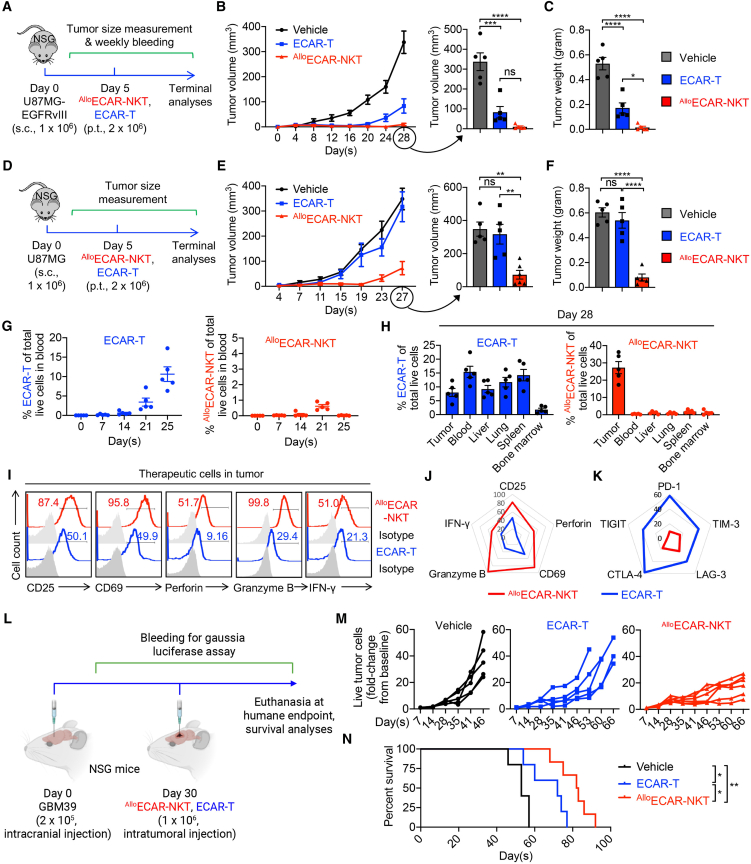
Allogeneic ECAR-NKT cells exhibit superior antitumor efficacy in human GBM xenograft mouse models (A–C) Study of *in vivo* antitumor efficacy of ^Allo^ECAR-NKT cells using a U87MG-EGFRvIII human GBM xenograft mouse model. (A) Experimental design. p.t., paratumoral injection; s.c., subcutaneous injection. (B) Tumor size measurements over time (*n* = 5). (C) Tumor weight measurements on day 28 (*n* = 5). (D–F) Study of *in vivo* antitumor efficacy of ^Allo^ECAR-NKT cells using a U87MG human GBM xenograft mouse model. Note that this tumor model recapitulates CAR antigen escape observed in GBM tumor cells. (D) Experimental design. (E) Tumor size measurements over time (*n* = 5). (F) Tumor weight measurements on day 27 (*n* = 4). (G and H) Studying the *in vivo* pharmacokinetics of ^Allo^ECAR-NKT cells. (G) FACS analyses of ^Allo^ECAR-NKT and ECAR-T cell percentage in mouse peripheral blood over time (*n* = 5). (H) FACS analyses of ^Allo^ECAR-NKT and ECAR-T cell percentage in the indicated tissues at the terminal day (day 28) (*n* = 5). (I–K) Studying the phenotype of ^Allo^ECAR-NKT cells in the TME. (I) FACS detection of the effector molecule expression in therapeutic cells collected from tumor sites of experimental mice on day 28. (J) Radar plot showing the effector molecule expression in therapeutic cells (*n* = 5). (K) Radar plot showing the immune checkpoint expression in therapeutic cells (*n* = 5). (L–N) Study of *in vivo* antitumor efficacy of ^Allo^ECAR-NKT cells using a GBM39 human GBM orthotopic xenograft mouse model. GBM39 is a neurosphere line derived from a primary GBM patient. (L) Experimental design. (M) Gaussia luciferase analyses showing the intracranial tumor burden in the experimental mice over time (*n* = 5–6). (N) Kaplan-Meier survival curves (*n* = 5–6). Representative of 2 (L–N) and 3 (A–K) experiments. Data are presented as the mean ± SEM. ns, not significant, ∗*p* < 0.05, ∗∗*p* < 0.01, ∗∗∗*p* < 0.001, ∗∗∗∗*p* < 0.0001, by 1-way ANOVA (B, C, E, and F), or log rank (Mantel-Cox) text adjusted for multiple comparisons (N).

We next evaluated the *in vivo* antitumor efficacy of ^Allo^ECAR-NKT cells using a series of human GBM xenograft mouse models. These models included both s.c. (Figures 4A, 4D, and S5A) and intracranial injections (Figure 4L) of GBM tumor cells, using either established tumor cell lines (Figures 4A, 4D, and S5A) or patient-derived neurosphere lines (Figure 4L). Importantly, both EGFRvIII^+^ (Figures 4A and S5A) and EGFRvIII^−^ tumor cells (Figure 4D) were employed; the EGFRvIII^−^ models served to mimic CAR antigen escape, a common mechanism of resistance observed following conventional CAR-T cell therapy.^10^ In all GBM models, therapeutic cells were administered either paratumorally or intracranially, mimicking clinical trial protocols where CAR-T cells are delivered via intraventricular administration.^53^^,^^54^ This localized delivery approach is designed to enhance therapeutic efficacy while minimizing systemic toxicities, such as cytokine release syndrome (CRS).^5^^,^^55^

In the first U87MG-EGFRvIII s.c. xenograft mouse model, both conventional ECAR-T and ^Allo^ECAR-NKT cells were able to suppress tumor growth (Figures 4A–4C). However, ^Allo^ECAR-NKT cells demonstrated significantly greater antitumor activity, as evidenced by markedly reduced tumor sizes and weights, with tumors becoming nearly undetectable (Figures 4A–4C). A dose-gradient comparison further demonstrated a clear dose-dependent antitumor response for both cell types, with higher doses leading to improved tumor control (Figures S5A–S5D). Notably, across all tested doses, ^Allo^ECAR-NKT cells consistently outperformed ECAR-T cells in tumor cell killing efficacy (Figures S5A–S5D).

In contrast, when GBM tumor cells lost EGFRvIII expression, conventional ECAR-T cells failed to control tumor growth, whereas ^Allo^ECAR-NKT cells retained potent tumor-suppressive capacity (Figures 4D–4F). This superior efficacy is likely attributed to the multifaceted tumor-targeting mechanisms of ^Allo^ECAR-NKT cells, particularly their ability to recognize tumor cells through NKR-mediated pathways (Figure 3). This unique feature enables ^Allo^ECAR-NKT cells to effectively target heterogeneous GBM tumors and overcome antigen escape, conferring a major therapeutic advantage over conventional CAR-T cell approaches.

Further pharmacokinetic analyses of the 2 therapeutic cell types revealed distinct *in vivo* distribution profiles. Weekly blood sampling showed that ^Allo^ECAR-NKT cells were largely absent from peripheral circulation throughout the study, whereas ECAR-T cells began to appear in the blood approximately 20 days post-injection (Figures 4G and S5E). Terminal tissue analysis demonstrated that ^Allo^ECAR-NKT cells remained predominantly localized at the tumor site, with minimal dissemination to peripheral organs (Figures 4H and S5F). In contrast, conventional ECAR-T cells exhibited broader tissue distribution, including detectable levels in the blood, spleen, lung, and liver (Figures 4H and S5F). These findings suggest that ^Allo^ECAR-NKT cells exhibit strong tumor-retention capacity, enabling focused cytotoxic activity at the tumor site while minimizing off-target migration. Conversely, ECAR-T cells display a more systemic trafficking pattern, potentially increasing exposure to non-tumor tissues. This divergence in biodistribution may be attributed to differences in chemokine receptor expression, which influence the migratory behavior and tissue homing properties of the respective cell types.^15^^,^^16^

We then compared the phenotypic profiles of the 2 therapeutic cell types within the TME using flow cytometry. Compared to conventional ECAR-T cells, ^Allo^ECAR-NKT cells exhibited a higher expression of T cell activation markers, including CD25 and CD69, as well as increased production of proinflammatory cytokines such as IFN-γ and elevated levels of cytotoxic molecules, including perforin and granzyme B (Figures 4I and 4J). Furthermore, ^Allo^ECAR-NKT cells expressed lower levels of T cell exhaustion markers, including PD-1, CTLA-4, TIM-3, LAG-3, and TIGIT (Figure 4K), which are well-studied immune checkpoint molecules.^56^^,^^57^^,^^58^ These findings suggest that ^Allo^ECAR-NKT cells maintain a more activated and functionally potent phenotype with reduced exhaustion in the TME, potentially contributing to their enhanced antitumor efficacy.

In another model, a GBM patient-derived neurosphere cell line was intracranially injected into the brains of NSG mice, establishing brain tumors over a 30-day period (Figure 4L). This model more accurately recapitulates the clinical disease setting in GBM patients. Therapeutic cells were administered via intracranial injection, mimicking the localized delivery approach used in clinical trials.^53^^,^^54^ Notably, ^Allo^ECAR-NKT cells demonstrated superior antitumor efficacy compared to conventional ECAR-T cells, as reflected by significantly reduced tumor burden and prolonged overall survival (Figures 4M and 4N).

Together, these *in vivo* models underscore the enhanced therapeutic potential of ^Allo^ECAR-NKT cells in treating GBM, particularly in the context of tumor heterogeneity and CAR antigen escape. Their ability to localize effectively to the tumor site, maintain robust cytotoxic and Th1 functional activity, and resist exhaustion in the immunosuppressive TME provides a multifaceted advantage over conventional CAR-T cells. These findings support the translational advancement of ^Allo^ECAR-NKT cells as a promising off-the-shelf, next-generation immunotherapy for patients with GBM and potentially other solid tumors with similar immunologic barriers.

### Allogeneic ECAR-NKT cells do not induce systemic toxicity in mouse models

Toxicities such as CRS and neurotoxicity are common adverse events associated with conventional CAR-T cell therapy, particularly in GBM, due to the abundance of monocytes and microglia in the brain.^59^^,^^60^^,^^61^^,^^62^ Upon activation in the TME, these myeloid cells can become proinflammatory, contributing to treatment-associated toxicities. To assess the safety profile of ^Allo^ECAR-NKT cells, we conducted a series of preclinical evaluations focused on their potential to induce CRS and long-term toxicity.

**Figure 5:**
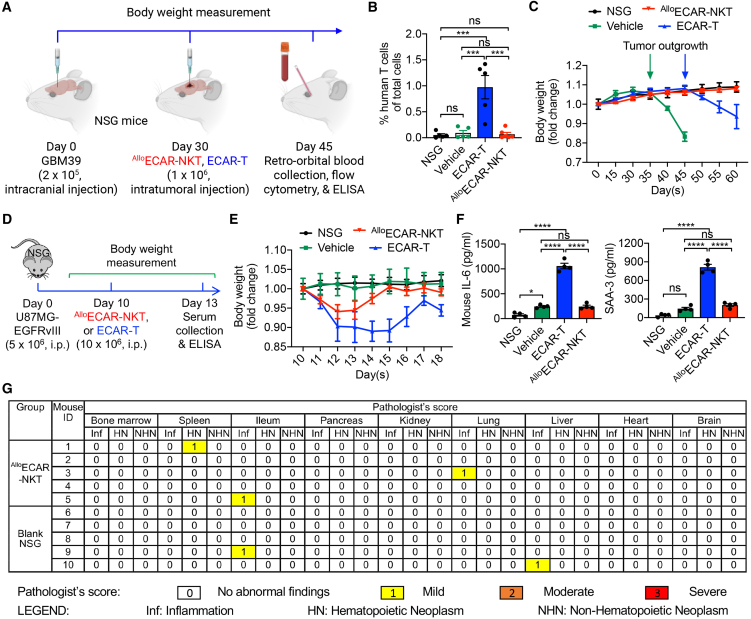
Allogeneic ECAR-NKT cells do not induce systemic toxicity in mouse models (A–C) Study of the *in vivo* safety of ^Allo^ECAR-NKT cells in a human GBM xenograft model when delivered through intracranial injections. (A) Experimental design. (B) FACS detection of human T cells (gated as human CD45^+^CD3^+^ cells) in blood collected from experimental mice at day 45. NSG indicates the tumor-free NSG mice (*n* = 5–6). (C) Body weight measurements over time (*n* = 5–6). (D–F) Study of the *in vivo* safety of ^Allo^ECAR-NKT cells in a human GBM xenograft model. (D) Experimental design. (E) Body weight measurements over time (*n* = 4). (F) ELISA analyses of mouse IL-6 and SAA-3 in mouse serum (*n* = 4). (G) Study of the long-term safety of ^Allo^ECAR-NKT cells using a human xenograft NSG mouse model. Tissues from experimental mice were collected 120 days after injection with ^Allo^ECAR-NKT cells. Data were presented as pathologist’s scores of individual mouse tissues (*n* = 5). Representative of 1 (G) or 2 (A–F) experiments. Data are presented as the mean ± SEM. ns, not significant, ∗*p* < 0.05, ∗∗∗*p* < 0.001, ∗∗∗∗*p* < 0.0001, by 1-way ANOVA (B and F).

In the intracranial human GBM xenograft mouse model, retro-orbital blood analysis revealed the presence of conventional ECAR-T cells in the peripheral blood, indicating that a portion of these cells could cross the blood-brain barrier (BBB) despite intracranial administration (Figures 5A and 5B). In contrast, ^Allo^ECAR-NKT cells were undetectable in the peripheral blood, suggesting superior brain localization with minimal leakage into the circulation, which is an important indicator of enhanced safety (Figures 5A and 5B). Furthermore, animals treated with ^Allo^ECAR-NKT cells maintained stable body weight throughout the study period until tumor progression, further supporting a favorable safety profile (Figure 5C). Importantly, due to their ability to target CD1d-expressing MDSCs and microglia within the GBM TME, ^Allo^ECAR-NKT cells not only enhance antitumor efficacy but also significantly reduce the population of proinflammatory myeloid cells (Figures 3Q–3T). This dual functionality contributes to lowering the risk of neurotoxicity, a common and serious side effect of conventional CAR-T cell therapies in GBM.^62^

CRS is a major concern in CAR-T cell therapy due to its potential to cause severe adverse effects.^59^^,^^60^^,^^61^^,^^62^ We first measured serum levels of mouse IL-6 and serum amyloid A-3 (SAA-3), 2 key biomarkers associated with CRS,^63^^,^^64^ using blood samples collected on day 45 and analyzed via enzyme-linked immunosorbent assay (ELISA) (Figure 5A). Interestingly, both CRS-related markers were detected at low levels in mice treated with either ECAR-T or ^Allo^ECAR-NKT cells (Figure S6A). This likely reflects the relatively low number of therapeutic cells administered in this model and the limited leakage of cells from the brain tumor site into the systemic circulation, thereby minimizing the induction of CRS (Figure 5B).

To more effectively evaluate CRS potential, we employed an alternative human tumor xenograft model involving intraperitoneal (i.p.) injection of both tumor and therapeutic cells.^63^^,^^64^ This approach facilitates enhanced interaction between the therapeutic cells and tumor cells within a confined anatomical compartment, thereby increasing the likelihood of inducing CRS and enabling its detection. Notably, previous studies using human tumor xenograft mouse models have shown that mouse macrophages can significantly contribute to the exacerbation of CRS.^63^^,^^64^ In a U87MG-EGFRvIII human GBM xenograft model, treatment with ^Allo^ECAR-NKT cells demonstrated a clear safety advantage over ECAR-T cells, as evidenced by more stable body weight and reduced levels of CRS-associated biomarkers (i.e., mouse IL-6 and SAA-3) in the serum (Figures 5D–5F). In addition, both ECAR-T and ^Allo^ECAR-NKT cells secreted high levels of human IFN-γ, reflecting their potent antitumor activity and shared Th1 functional phenotype (Figure S6B). However, ECAR-T cells produced detectable levels of human IL-6, whereas ^Allo^ECAR-NKT cells secreted minimal IL-6, further supporting their favorable safety profile (Figure S6B). These findings suggest that ^Allo^ECAR-NKT cells may pose a lower risk of CRS-like responses, potentially due to their innate-like NK cell properties and their ability to counteract macrophage-mediated inflammatory amplification (Figures 3H, 3I, and 3Q–3T).^15^^,^^33^

Remarkably, ^Allo^ECAR-NKT cells demonstrated sustained safety, as evidenced by minimal organ toxicity observed up to 120 days post-adoptive transfer in NSG mice (Figure 5G). Furthermore, due to their invariant TCR recognizing the non-polymorphic major histocompatibility complex-like molecule CD1d, ^Allo^ECAR-NKT cells did not induce GvHD (Figures S6C–S6E).^65^^,^^66^^,^^67^ In contrast, conventional ECAR-T cells triggered severe GvHD manifestations (Figures S6C–S6E). These findings collectively highlight the favorable safety profile of ^Allo^ECAR-NKT cells and support their potential as an off-the-shelf therapeutic strategy for the treatment of GBM.

## Discussion

Here, we report the development of an allogeneic HSPC-engineered EGFRvIII-specific CAR-NKT cell therapy for the treatment of GBM, characterized by high yield, high purity, potent antitumor efficacy, and a favorable safety profile. Utilizing our established HSPC engineering and *ex vivo* differentiation platform, we successfully generated clinically relevant, scalable ^Allo^ECAR-NKT cells that meet key requirements for off-the-shelf allogeneic cell therapy, including high manufacturing consistency, purity, VCN within US Food and Drug Administration-mandated limits, and robust product yields across multiple CB donors (Figure 1).

Importantly, we observed no significant variability in phenotypic or functional characteristics among ^Allo^ECAR-NKT cell products generated from different CB donors or engineered with diverse constructs (e.g., with or without IL-15 or different CARs) (Figure 1),^15^^,^^16^ highlighting the robustness and reproducibility of our platform. This versatility enables facile adaptation of the platform for additional CAR targets relevant to GBM, such as IL-13 receptor subunit α-2 (IL-13Rα2), human epidermal growth factor receptor 2, and disialoganglioside GD2, which have previously demonstrated clinical relevance in CAR-T cell therapies for GBM.^8^^,^^62^^,^^68^^,^^69^^,^^70^ Furthermore, our approach supports flexible multiplex gene engineering to enhance immune functionality. Beyond IL-15, ^Allo^ECAR-NKT cells can be engineered to express other immunomodulatory genes, including chemokines (e.g., CXCL9, CXCR6), cytokines (e.g., IL-12, IL-18, IL-21), and metabolic regulators (e.g., peroxisome proliferator-activated receptor-γ-coactivator 1-α), enabling further customization of the therapeutic phenotype to overcome the immunosuppressive TME.^5^^,^^62^^,^^71^^,^^72^^,^^73^^,^^74^ Overall, our platform offers a robust, versatile, and clinically scalable approach for generating optimized ^Allo^ECAR-NKT cells, supporting their potential as a next-generation immunotherapy for GBM.

The challenges to effective CAR-T cell therapy for GBM remain significant and include the identification of optimal tumor-associated antigens, the antigenic heterogeneity of GBM tumors, subsequent tumor antigen escape, limited T cell trafficking and infiltration into the tumor bed, the highly immunosuppressive TME, and the toxicity.^10^^,^^55^^,^^62^^,^^75^^,^^76^ In this study, we performed direct comparative analyses between ^Allo^ECAR-NKT cells and conventional ECAR-T cells using multiple *in vitro* assays and *in vivo* human GBM xenograft models. Our data demonstrate that ^Allo^ECAR-NKT cells effectively address these key limitations by leveraging both CAR-dependent and innate-like mechanisms to target heterogeneous tumor cells, displaying focused tumor homing, overcoming TME-mediated immunosuppression, and demonstrating a favorable safety profile. It is important to note that ^Allo^ECAR-NKT cells, as a new allogeneic cell product, share mechanisms of action similar to those of PBMC-derived CAR-NKT cells.^21^^,^^47^^,^^77^^,^^78^^,^^79^^,^^80^ These include dual cytotoxic functionality characteristic of both T cells and NK cells, as well as the capacity to target the TME via CD1d recognition. Our primary objective in this study was to apply this platform to develop a clinically translatable allogeneic CAR-NKT cell therapy, with an emphasis on advancing its scalable manufacturing and therapeutic application. Future studies will focus on further elucidating additional mechanisms of action mediated by ^Allo^ECAR-NKT cells.

Antigenic heterogeneity in GBM and tumor antigen escape following CAR-T cell therapy represent major barriers to effective treatment. GBM are characterized by significant cellular and molecular diversity, often resulting in partial or complete loss of target antigen expression and subsequent tumor recurrence.^62^ This resistance mechanism has been well documented in GBM patients in clinical trials, where recurrent tumors frequently downregulate or lose the expression of the cognate antigen following treatment with CAR-T cells targeting IL-13Rα2 or EGFRvIII.^7^^,^^81^ Notably, our ^Allo^ECAR-NKT cells exhibited superior tumor-targeting capabilities by leveraging both CAR-mediated recognition and multiple NKR-mediated pathways (Figures 3 and 4). These NKRs, such as NKG2D, DNAM-1, NKp30, and others, enable recognition of a broad range of ligands commonly expressed on GBM tumor cells, including ULBPs, MICA/B, CD112, and CD155 (Figures 3D, 3H, and 3I). This multi-receptor targeting approach allows ^Allo^ECAR-NKT cells to engage tumor cells through diverse mechanisms, thereby enhancing cytotoxic efficacy and reducing the likelihood of immune escape. Indeed, ^Allo^ECAR-NKT cells effectively targeted and killed EGFRvIII-low and EGFRvIII^−^ GBM cells both *in vitro* and *in vivo*, outperforming conventional ECAR-T cells (Figures 3 and 4). These results highlight the unique advantages of ^Allo^ECAR-NKT cells in addressing the challenge of antigen escape and underscore their therapeutic potential in treating heterogeneous and treatment-resistant GBM.

Intracranial delivery of CAR-T cells has emerged as an effective strategy for treating GBM, enabling localized administration, direct tumor engagement, and reduced systemic exposure, thus enhancing both efficacy and safety.^54^^,^^55^^,^^69^ Given these advantages, intracranial administration is a promising route not only for conventional CAR-T cell therapy but also for allogeneic cell products, such as our ^Allo^ECAR-NKT cells. In a notable Phase 1 clinical trial, Brown et al. evaluated the locoregional delivery of IL-13Rα2-targeting CAR-T cells in 65 patients with recurrent high-grade glioma.^69^ The study demonstrated the safety, feasibility, and determination of the maximum tolerated dose as primary endpoints. Importantly, IL-13Rα2-specific CAR-T cells were detected in the cerebrospinal fluid and tumor cavity fluid, and were capable of trafficking from the central nervous system to the peripheral circulation, raising concerns regarding potential systemic toxicity.^69^ Similarly, in our preclinical studies, we observed that conventional ECAR-T cells, even when administered intracranially, were able to cross the BBB and enter the peripheral circulation. In contrast, ^Allo^ECAR-NKT cells remained localized within the brain, with minimal leakage into the periphery (Figures 5A–5C). This enhanced localization may be attributed to the unique chemokine receptor expression profile of HSPC-derived CAR-NKT cells and their innate preference for tissue residency.^15^^,^^24^ Overall, ^Allo^ECAR-NKT cells exhibited superior tumor-killing efficacy while maintaining focused tumor homing, highlighting their potential as a safe and effective intracranial immunotherapy for GBM.

The GBM TME is a complex and dynamic milieu that surrounds and interacts with tumors, fostering immunosuppressive conditions that impede effective antitumor immunity. In GBM, the TME is characterized by the accumulation of immunoregulatory cell populations, including regulatory T cells, MDSCs, and TAMs, which collectively suppress cytotoxic lymphocyte activity.^10^^,^^82^^,^^83^ Moreover, intratumoral hypoxia, a hallmark of the GBM microenvironment, further contributes to immune evasion by impairing immune cell function via upregulation of hypoxia-inducible factor 1-α.^51^^,^^52^^,^^84^^,^^85^ Overcoming the immunosuppressive TME is therefore essential for enhancing the efficacy of cell-based immunotherapies. To address this challenge, several strategies have been explored, such as engineering CAR-T cells to secrete proinflammatory cytokines (e.g., IL-12, IL-18, IL-21), expressing dominant-negative receptors for inhibitory molecules (e.g., dominant-negative transforming growth factor β [TGF-β] receptors), or designing dual-specific CARs that simultaneously target tumor-associated antigens and immunosuppressive mediators (e.g., TGF-β).^86^^,^^87^^,^^88^ In our study, we demonstrated that ^Allo^ECAR-NKT cells can directly target immunosuppressive myeloid cells, including TAMs, through their invariant NKT TCRs, which specifically recognizes CD1d, a molecule highly expressed on these suppressive cell types (Figures 3Q–3T and S4C–S4G).^50^^,^^89^ This CD1d-mediated recognition and cytotoxicity are unique to NKT cells and represent a key advantage over conventional CAR-T cells. Our findings are supported by prior studies in other tumor models, further emphasizing the superior capacity of CAR-NKT cells to simultaneously target GBM tumor cells and remodel the immunosuppressive TME.^80^^,^^90^^,^^91^^,^^92^^,^^93^

Common toxicities associated with CAR-T cell therapy include CRS, immune effector cell-associated neurotoxicity syndrome, tumor lysis syndrome, and acute anaphylaxis.^59^^,^^73^^,^^94^ Among these, CRS is the most frequently observed and is primarily driven by the excessive release of proinflammatory cytokines such as IL-6, IFN-γ, and TNF-α. Clinically, CRS presents with symptoms that include fever, hypotension, tachycardia, hypoxia, and, in severe cases, multiorgan dysfunction.^59^^,^^73^^,^^94^ Notably, prior studies have demonstrated that interactions between CAR-T cells and components of the myeloid compartment, particularly macrophages and monocytes, can exacerbate CRS.^63^^,^^64^ In the context of GBM, where the TME is enriched with MDSCs and TAMs, the risk of severe CRS is amplified further. Importantly, in our study, ^Allo^ECAR-NKT cells exhibited a significantly reduced risk of CRS compared to conventional ECAR-T cells in a previously validated humanized mouse model (Figures 5D–5F).^63^^,^^64^ This enhanced safety profile may be attributed to the intrinsic NK-like properties of NKT cells (Figures 2D and 2E), along with their ability to selectively eliminate proinflammatory myeloid populations, including monocytes and macrophages/microglia, via CD1d-restricted recognition (Figures 3Q–3T and S4C–S4G). These findings highlight the potential of ^Allo^ECAR-NKT cells to minimize CAR-T cell-associated toxicities, particularly in TME-rich malignancies such as GBM.

In addition, we acknowledge the potential safety concern associated with ^Allo^ECAR-NKT cell-mediated cytotoxicity against CD1d^+^ macrophages. While this activity contributes to remodeling the immunosuppressive TME through depletion of TAMs, it may also pose a theoretical risk of off-target effects against CD1d^+^ myeloid populations in healthy tissues. In our preclinical models, however, we observed no evidence of systemic toxicity or organ damage, suggesting that the cytotoxic activity of ^Allo^ECAR-NKT cells is preferentially localized to the TME (Figure 5). In future clinical applications, the allogeneic nature of ^Allo^ECAR-NKT cells may further mitigate long-term safety risks, as these cells are expected to exert antitumor effects within a defined therapeutic window before being rejected by the host immune system, a phenomenon observed with other allogeneic CAR-T cell platforms.^25^^,^^95^^,^^96^^,^^97^ Importantly, because allogeneic CAR-NKT cells do not target normal HSPCs, endogenous hematopoiesis, including the myeloid compartment, is preserved and can be reconstituted following therapy.^15^^,^^33^ Nevertheless, the potential for off-tumor effects should be carefully assessed in future translational and clinical studies to fully characterize the safety profile of ^Allo^ECAR-NKT therapy.

We also acknowledge that, unlike the ^Allo^ECAR-NKT cells, the conventional ECAR-T cells used in this study were not engineered to secrete IL-15. A future comparison between ^Allo^ECAR-NKT cells and IL-15-armored ECAR-T cells would be valuable to further delineate the contribution of IL-15 to therapeutic efficacy and persistence. IL-15 has been shown to significantly enhance CAR-T cell expansion, persistence, and antitumor activity in multiple preclinical models, and recent clinical studies, such as the trial using IL-15-engineered GPC3-targeting CAR-T cells for hepatocellular carcinoma, have demonstrated promising outcomes.^98^ However, these IL-15-enhanced CAR-T therapies have also raised safety concerns, particularly the induction of systemic CRS.^98^ In contrast, ^Allo^ECAR-NKT cells demonstrated a favorable safety profile in our study (Figure 5), highlighting their potential advantage for clinical translation and development.

Altogether, the development of ^Allo^ECAR-NKT cells represents a compelling step toward the realization of safe, effective, and off-the-shelf immunotherapy for GBM. By uniting the precision of CAR engineering with the innate adaptability of NKT cells, this approach offers a uniquely powerful means to navigate the complex tumor landscape of GBM. With a clinically adaptable manufacturing pipeline and a strong safety-efficacy balance, ^Allo^ECAR-NKT cell therapy holds immense translational promise and stands poised to redefine the therapeutic paradigm for patients with GBM and other hard-to-treat solid tumors.

## Materials and methods

### Study approval

Animal studies were conducted under protocols approved by the University of California, Los Angeles (UCLA) Division of Laboratory Animal Medicine. Healthy donor PBMCs were obtained from the UCLA/Center for AIDS Research (CFAR) Virology Core Laboratory and HemaCare under informed consent and in compliance with federal and state regulations; no identifying information was provided. All patient-derived tumor tissue was obtained through the UCLA institutional review board (IRB) protocol 10–000655, after written informed consent was obtained from patients.

### Mice

NOD.Cg-Prkdc^SCID^ Il2rg^tm1Wjl^/SzJ (NOD/SCID/IL-2Rγ^−/−^, NSG) mice were maintained in the animal facilities of UCLA under the following housing conditions: temperature ranging from 68°F to 79°F, humidity maintained at 30%–70%, a light cycle of On at 6:00 a.m. and Off at 6:00 p.m., and room pressure set to negative. Six- to 10-week-old male or female mice were used for all experiments unless otherwise indicated. Sex was not considered in the study design and analysis, as no significant differences were observed in the human GBM NSG mouse models used. All animal experiments were approved by the Institutional Animal Care and Use Committee of UCLA. All mice were bred and maintained under specific pathogen-free conditions, and all experiments were conducted in accordance with the animal care and use regulations of the Division of Laboratory Animal Medicine at UCLA. Experimental mice were randomly assigned to treatment groups to avoid statistically significant differences in the baseline tumor burden.

### Media and reagents

The X-VIVO 15 Serum-Free Hematopoietic Cell Medium (catalog no. 04418Q) was purchased from Lonza. The StemSpan T cell Generation Kit (catalog no. 09940), comprising the StemSpan SFEM II Medium (catalog no. 09605), the StemSpan Lymphoid Progenitor Expansion Supplement (catalog no. 09915), the StemSpan LPMS (catalog no. 09930), the StemSpan Lymphoid Progenitor Differentiation Coating Material (catalog no. 09925), and the ImmunoCult Human CD3/CD28/CD2 T Cell Activator (catalog no. 10970), and MethoCult H4330 Methylcellulose-Based Medium (catalog no. 04330) were purchased from STEMCELL Technologies. The CTS OpTmizer T-Cell Expansion SFM (no phenol red, bottle format, catalog no. A3705001), the RPMI 1640 cell culture medium (catalog no. MT10040CV), and the DMEM cell culture medium (catalog no. MT10013CV) were purchased from Thermo Fisher Scientific. The CryoStor Cell Cryopreservation Media CS10 (catalog no. C2874) and Iscove’s modified Dulbecco’s medium (catalog no. I3390) were purchased from MilliporeSigma. The C10 medium was made of RPMI 1640 cell culture medium, supplemented with fetal bovine serum (FBS; 10% v/v), penicillin/streptomycin/glutamine (P/S/G; 1% v/v), MEM nonessential amino acids (NEAA) (1% vol/vol), HEPES (10 mM), sodium pyruvate (1 mM), β-mercaptoethanol (β-ME) (50 μM), and Normocin (100 μg/mL). The homemade D10 medium was made of DMEM supplemented with FBS (10% v/v), P/S/G (1% v/v), and Normocin (100 μg/mL). The homemade R10 medium was made of RPMI 1640 supplemented with FBS (10% v/v), P/S/G (1% v/v), and Normocin (100 μg/mL).

αGC (KRN7000, catalog no. 867000) was purchased from Avanti Polar Lipids. Recombinant human IL-2 (catalog no. 200-02), IL-3 (catalog no. 200-03), IL-7 (catalog no. 200-07), IL-15 (catalog no. 200-15), IL-21 (catalog no. 200-21), IFN-γ (catalog no. 300-02), Flt3 ligand (Flt3L, catalog no. 300-19), macrophage-colony-stimulating factor (M-CSF, catalog no. 300-25), stem cell factor (SCF, catalog no. 300-07), and thrombopoietin (TPO, catalog no. 300-18) were purchased from PeproTech. FBS (lot no. 2087050) was purchased from GIBCO and β-ME (catalog no. 1610710) was purchased from Bio-Rad. P/S/G (catalog no. 10-378-016), MEM NEAA (catalog no. 11-140-050), HEPES Buffer Solution (catalog no. 15630080), and sodium pyruvate (catalog no. 11360070) were purchased from GIBCO. Normocin was purchased from InvivoGen (catalog no. NC9390718).

### Lentiviral vectors

A parental lentivector, pMNDW, was utilized to construct the lentiviral vectors employed in this study.^19^^,^^20^ The 2A sequences derived from foot-and-mouth disease virus (F2A), porcine teschovirus-1 (P2A), and thosea asigna virus (T2A) were used to link the inserted genes to achieve co-expression. The Lenti/iNKT-ECAR-IL-15 vector was generated by inserting into the pMNDW parental backbone a synthetic tetracistronic gene encoding human iNKT TCRα-F2A-iNKT TCRβ-P2A-ECAR-T2A-IL-15 (ECAR denotes an EGFRvIII-specific CAR,^7^ and IL-15 represents the secreted form of human IL-15). The Lenti/iNKT-BCAR-IL-15 vector was generated by inserting into the pMNDW parental backbone a synthetic tetracistronic gene encoding human iNKT TCRα-F2A-iNKT TCRβ-P2A-BCAR-T2A-IL-15 (BCAR denotes a BCMA-specific CAR).^15^ The Lenti/iNKT vector was constructed by inserting a synthetic bicistronic gene encoding human iNKT TCRα-F2A-iNKT TCRβ into pMNDW. The Lenti/ECAR vector was constructed by inserting a synthetic gene encoding ECAR into pMNDW. The Lenti/FG vector was generated by inserting a synthetic bicistronic gene encoding Fluc-P2A-EGFP into the pMNDW backbone. The Lenti/EGFRvIII vector was constructed by inserting a synthetic gene encoding human EGFRvIII into pMNDW. All synthetic gene fragments were obtained from GenScript and Integrated DNA Technologies. Lentiviral particles were generated utilizing HEK293T cells by employing a standardized transfection procedure with the Trans-IT-Lenti Transfection Reagent (Mirus Bio).^19^^,^^20^ Subsequently, a concentration protocol was applied using Amicon Ultra Centrifugal Filter Units in accordance with the manufacturer’s specifications (MilliporeSigma).

### Stable cell lines

Human GBM cell line U87MG (catalog no. HTB-14) was purchased from the American Type Culture Collection (ATCC). To establish stable tumor cell lines that overexpress firefly luciferase and EGFP FG, the parental tumor cell lines were transduced with lentiviral vectors carrying the specific genes of interest (i.e., Lenti/FG). At 72 h after lentiviral transduction, the cells underwent flow cytometry sorting to isolate the genetically modified cells (identified as GFP^+^ cells) necessary for creating stable cell lines. The artificial antigen-presenting cell line (aAPC) was generated by engineering the K562 human chronic myelogenous leukemia cell line (ATCC, catalog no. CCL-243) to overexpress human CD80/CD83/CD86/41BBL co-stimulatory receptors. The aAPC-EGFRVIII cell lines were generated by further engineering the parental aAPC line to overexpress human EGFRvIII.

### Primary GBM patient-derived neurosphere cell lines

Tumor resections were mechanically and enzymatically dissociated using the Miltenyi Biotec Human Tumor Dissociation Kit (catalog no. 130-094-929) within 6 h of surgery, followed by removal of red blood cells with ACK lysis buffer (GIBCO, catalog no. A10492-01). Next, antibody-conjugated magnetic beads were used to remove CD45^+^ cells (Miltenyi Biotec, catalog no. 130-045-801) and myelinated cells (Miltenyi Biotec, catalog no. 130-096-433) by performing column-based filtrations. Primary GBM cells were established and cultured as gliomaspheres in media consisting of DMEM/F12 (GIBCO, catalog no. 11330032), B27 (Invitrogen, catalog no. 12587010), P/S (Invitrogen, catalog no. 15140122), and GlutaMAX (Invitrogen, catalog no. 35050061) supplemented with heparin (5 mg/mL, Sigma, catalog no. H3149), EGF (20 ng/mL, Gibco, catalog no. PHG0313), and fibroblast growth factor (20 ng/mL, GIBCO, catalog no. PHG0263). When passaged, gliomaspheres were dissociated to single-cell suspensions with TrypLE (Thermo Fisher, catalog no. 12605028). All cells were grown under 37°C and 5% CO_2_ and were routinely monitored and tested negative for the presence of mycoplasma with a commercially available kit (MycoAlert, Lonza). Gliomasphere cell lines were used at fewer than 15 passages. All cells were authenticated by short tandem repeat analysis. These tumor cells were stably transduced with secreted Gaussia luciferase (sGluc)-encoding reporter gene (pLenti_CMV_GLuc_T2A_EGFP plasmid, Prolume) to enable non-invasive and routine quantification of tumor burden *in vivo*.

### Human CD34^+^ HSPCs and PBMCs

Purified human CD34^+^ HSPCs derived from CB were purchased from HemaCare. Healthy donor PBMCs were provided by the UCLA/CFAR Virology Core Laboratory without identification information under federal and state regulations. Upon receipt, both HSPCs and PBMCs were promptly aliquoted and cryopreserved in liquid nitrogen for subsequent experimental use.

### Antibodies and flow cytometry

Fluorochrome-conjugated antibodies specific for human CD45 (clone HI30; Peridinin-Chlorophyll-Protein Complex [PerCP], fluorescein isothiocyanate [FITC], or Pacific Blue conjugated, 1:500, catalog nos. 982318, 982316, or 982306), CD3 (clone HIT3a; Pacific Blue, phycoerythrin [PE], or PE-Cy7 conjugated, 1:500, catalog nos. 300330, 300308, or 300316), CD5 (clone UCHT2; PerCP conjugated, 1:200, catalog no. 300618), CD7 (clone CD7-6B7; APC conjugated, 1:200, catalog no. 343108), CD1d (clone 51.1; PE-Cy7 or APC conjugated, 1:50, catalog nos. 350310 or 350308), CD4 (clone OKT4; PE-Cy7, PerCP, or FITC conjugated, 1:500, catalog nos. 317414, 317432, or 317408), CD8A (clone SK1; PE, APC-Cy7, or APC conjugated, 1:300, catalog no. 344706, 344714, or 344722), CD8B (clone QA20A40; APC conjugated, 1:500, catalog no. 387305), CD14 (clone HCD14; Pacific Blue conjugated, 1:100, catalog no. 367122), CD19 (clone HIB19; APC-Cy7 conjugated, 1:200, catalog no. 302218), CD34 (clone 581; PerCP conjugated, 1:500, catalog no. 343520), CD31 (clone WM59; FITC conjugated, 1:100, catalog no. 989002), CD69 (clone FN50; PE-Cy7 or PerCP conjugated, 1:50, catalog nos. 310912 or 310928), EGFRvIII (clone WM53; APC or PE conjugated, 1:50, catalog nos. 355109 or 355104), CD107a (clone H4A3; FITC conjugated, 1:200, catalog no. 338606), CD112 (clone TX31; PE conjugated, 1:250, catalog no. 337410), CD155 (clone SKII.4; PE-Cy7 conjugated, 1:250, catalog no. 337614), CD11b (clone ICRF44; FITC conjugated, 1:500, catalog no. 982614), MICA/MICB (clone 6D4; PE or APC conjugated, 1:25, catalog nos. 320906 or 320908), 41BBL (clone 5F4; PE conjugated, 1:500, catalog no. 311504), CD83 (clone HB15e; APC-Cy7 conjugated, 1:500, catalog no. 305330), CD86 (clone IT2.2; APC conjugated, 1:500, catalog no. 305412), PD-1 (clone A17188A; PE or FITC conjugated, 1:25, catalog nos. 379210 or 379206), TIM-3 (clone A18087E; APC conjugated, 1:25, catalog no. 364804), CTLA-4 (clone L3D10; APC conjugated, 1:50, catalog no. 369606), TIGIT (clone A15153G; PE conjugated, 1:50, catalog no. 372706), LAG-3 (clone 7H2C65; PE-Cy7 conjugated, 1:25, catalog no. 369208), NKG2D (clone 1D11; PE-Cy7 conjugated, 1:50, catalog no. 320812), DNAM-1 (clone 11A8; APC conjugated, 1:50, catalog no. 338312), NKp30 (clone P30-15; APC conjugated, 1:50, catalog no. 325210), NKp46 (clone 9E2, PE conjugated, 1:50, catalog no. 331908), IFN-γ (clone B27; PE-Cy7 conjugated, 1:50, catalog no. 506518), granzyme B (clone QA16A02; APC conjugated, 1:2,000 or 1:5,000, catalog no. 372204), perforin (clone dG9; PE-Cy7 conjugated, 1:50 or 1:100, catalog no. 308126), TNF-α (clone MAb11; APC conjugated, 1:4,000, catalog no. 502912), IL-2 (clone MQ117H12; APC-Cy7 conjugated, 1:50, catalog no. 500342), β2-microglobulin (B2M) (clone 2M2; FITC or APC conjugated, 1:2,000 or 1:5,000, catalog nos. 316304 or 316311), HLA-DR (clone L243; APC-Cy7 conjugated, 1:200 or 1:500, catalog no. 307618), HLA-DR/-DP/-DQ (clone Tü39; FITC conjugated, 1:200 or 1:500, catalog no. 361706), pSTAT5-phospho (clone A17016B.Rec; PE conjugated, 1:200, catalog no. 936904), and Bcl-2 (clone BCL/10C4; 1:200, catalog no. 633512) were purchased from BioLegend. Fluorochrome-conjugated antibodies specific for human iNKT TCR Vɑ24-Jβ18 (clone 6B11; PE conjugated, 1:20, catalog no. 552825) were purchased from BD Biosciences. Fluorochrome-conjugated antibodies specific for human fibroblast activation protein FAP (clone 427819; PE conjugated, 1:100, catalog no. FAB3715P), ULBP-1 (clone 170818; PE conjugated or unconjugated, 1:25, catalog nos. FAB1380P or MAB1380), and ULBP-2,5,6 (clone 165903; APC conjugated, 1:25, catalog no. FAB1298A) were purchased from R&D Systems. A goat anti-mouse immunoglobulin G F(ab′)2 secondary antibody (catalog no. A-11001) and Bcl-xL (clone 7B2.5; FITC conjugated, 1:200, catalog no. MA5-28637) were purchased from Thermo Fisher. Fixable Viability Dye eFluor506 (e506; 1:500, catalog no. 65-0866-14) was purchased from Affymetrix eBioscience. Mouse Fc Block (anti-mouse CD16/32, catalog no. 553141) was purchased from BD Biosciences, Human Fc Receptor Blocking Solution (TrueStain FcX) was purchased from BioLegend (catalog no. 422302). In our study, note the use of antibodies with identical clones but differing conjugated fluorochromes, with one typical antibody listed herein.

All fluorescence-activated cell sorting (FACS) staining was performed following the manufacturers’ provided protocols. Appropriate isotype staining controls were used for all staining procedures. Stained cells were analyzed using a MACSQuant Analyzer 10 flow cytometer (Miltenyi Biotech), following the manufacturer’s instructions. FlowJo software version 9 (BD Biosciences) was used for data analysis.

### ELISAs

The ELISAs for measuring human and mouse cytokines were conducted according to a standard protocol provided by BD Biosciences. Supernatants from cell culture experiments were collected and analyzed to quantify cytokines (e.g., human IFN-γ, TNF-α, IFN-γ, IL-2, IL-4, IL-6, and IL-15; mouse IL-6 and SAA-3). The capture and biotinylated antibodies used for cytokine detection were sourced from BD Biosciences, while the streptavidin-horseradish peroxidase conjugate was obtained from Invitrogen. Human and mouse cytokine standards were purchased from eBioscience, and the tetramethylbenzidine substrate was acquired from Thermo Scientific (catalog no. PI34021). Human IL-17A ELISA kits were purchased from Invitrogen (catalog no. BMS2017). Mouse SAA-3 ELISA kits were purchased from Millipore Sigma (catalog no. EZMSAA3). Absorbance of the samples was measured at 450 nm using an Infinite M1000 microplate reader (Tecan).

### Generation of HSPC-engineered ^Allo^ECAR-NKT cells

^Allo^ECAR-NKT cells were generated by differentiating gene-engineered human CB CD34^+^ HSPCs in a 5-stage clinically guided *ex vivo* HSPC-derived NKT cell culture method. The complete methodology and step-by-step protocols have been described in detail in previously published studies.^15^^,^^24^ Here, we provide a summary of the key steps involved in the culture and generation of ^Allo^ECAR-NKT cells. A key distinction in the current work includes the use of a lentiviral vector, Lenti/iNKT-ECAR-IL-15, specifically targeting EGFRvIII. At stage 4 of the culture, ^Allo^ECAR-NKT cells were stimulated using an aAPC-EGFRvIII approach, in place of the previously described antibody-based or αGC-loaded PBMC stimulation methods.^15^^,^^24^ This change was made due to the higher yield achieved with the aAPC-EGFRvIII approach. Additionally, we employed a confined virus titer during transduction, resulting in a VCN of 3–4 in the final cell product—falling within the safety range required for CAR-T cell therapy manufacturing.^74^^,^^99^

At stage 0, the frozen stock of human CD34^+^ HSPCs was thawed and cultured in T cell X-VIVO 15 Serum-Free Hematopoietic Stem Cell Medium supplemented with human Flt3L (50 ng/mL), SCF (50 ng/mL), TPO (50 ng/mL), and IL-3 (20 ng/mL) for 24 h. Lentiviral transduction was subsequently carried out for an additional 24 h using the Lenti/iNKT-ECAR-IL-15 vector.

At stage 1, transfected HSPCs harvested were cultured in the feeder-free StemSpan SFEM II Medium supplemented with StemSpan Lymphoid Progenitor Expansion Supplement for 14 days. HSPCs were cultured in CELLSTAR24-well Cell Culture Nontreated Multiwell Plates (VWR, catalog no. 82050-892). StemSpan Lymphoid Differentiation Coating Material (500 μL/well, diluted to a final concentration of 1× from a stock dilution of 100×) was applied to the plates and left for 2 h at room temperature or overnight at 4°C. Subsequently, 500 μL of the transfected CD34^+^ HSPC suspension, with a density of 2 × 10^4^ cells/mL, was added to each pre-coated well. Half of the medium in each well was removed and replaced with fresh medium twice per week.

At stage 2, the stage 1 cells were harvested and cultured in the feeder-free StemSpan SFEM II Medium supplemented with StemSpan Lymphoid Progenitor Maturation Supplement for ∼7 days. StemSpan Lymphoid Differentiation Coating Material (1 mL/well, diluted to a final concentration of 1×) was applied to Non-Treated Falcon Polystyrene 6-Well Microplates (Thermo Fisher Scientific, catalog no. 140675); 2 mL of the harvested stage 1 cells, resuspended with a density of 1 × 10^5^ cells/mL, was added into each pre-coated well. The cell density was maintained at 1–2 × 10^6^ cells per well during the stage 2 culturing. Cells were passaged 2–3 times per week, with the addition of fresh medium for each passage.

At stage 3, the stage 2 cells were harvested and cultured in the feeder-free StemSpan SFEM II Medium supplemented with StemSpan Lymphoid Progenitor Maturation Supplement, CD3/CD28/CD2 T Cell Activator, and human recombinant IL-15 (20 ng/mL) for ∼7 days. StemSpan Lymphoid Differentiation Coating Material (1 mL/well, diluted to a final concentration of 1×) was applied to Non-Treated Falcon Polystyrene 6-Well Microplates (Thermo Fisher Scientific, catalog no. 08-772-49); 2 mL of the harvested stage 2 cells, resuspended with a density of 5 × 10^5^ cells/mL, was added into each pre-coated well. The cell density was maintained at 1–2 × 10^6^ cells per well during the stage 3 culturing. Cells were passaged 2–3 times per week with the addition of fresh medium for each passage.

At stage 4, the stage 3 cells were harvested and verified by flow cytometry to confirm their status as mature ^Allo^ECAR-NKT cells or their derivatives; then, the cells underwent expansion stage via an aAPC-based expansion. aAPCs were irradiated at 10,000 rads using a Rad Source RS-2000 X-Ray Irradiator (Rad Source Technologies). The stage 3 mature ^Allo^ECAR-NKT cells and derivatives were co-cultured with the irradiated aAPCs (with a ratio of 1:1). The cells were resuspended in expansion medium (the CTS OpTmizer T cell Expansion Serum Free Medium [Thermo Fisher Scientific] or the homemade C10 medium) supplemented with human IL-7 (10 ng/mL) and IL-15 (10 ng/mL) at a density of 0.5–1 × 10^6^ cells/mL; 2 mL cell suspension was seeded into each well of the Corning Costar Flat Bottom Cell Culture 6-Well Plates. The cell density was maintained at 0.5–1 × 10^6^ cells/mL during the expansion stage. Cells were passaged 2–3 times per week with the addition of fresh medium for each passage. The expanded ^Allo^ECAR-NKT cells were aliquoted and cryopreserved in CryoStor Cell Cryopreservation Media CS10 using a Thermo Scientific CryoMed Controlled-Rate Freezer 7450 for stock.

### Generation of PBMC-derived conventional αβ T cells

PBMCs from healthy donors were utilized to generate conventional αβ T cells, referred to as PBMC-T cells. To produce PBMC-T cells, PBMCs were activated using Dynabeads Human T-Activator CD3/CD28 (Thermo Fisher Scientific, catalog no. 11131D) following the manufacturer’s guidelines. The activated cells were then cultured in C10 medium supplemented with 20 ng/mL IL-2 for a duration of 2–3 weeks.

### Generation of ECAR-T cells

PBMCs from healthy donors were utilized to generate conventional ECAR-T cells. To produce these cells, non-treated tissue culture 24-well plates (Corning, catalog no. 3738) were coated with Ultra-LEAF Purified Anti-Human CD3 Antibody (clone OKT3, BioLegend) at 1 μg/mL (500 μL/well) at room temperature for 2 h or at 4°C overnight. PBMCs were resuspended in the C10 medium supplemented with 1 μg/mL Ultra-LEAF Purified Anti-Human CD28 Antibody (clone CD28.2, BioLegend) and 30 ng/mL IL-2, followed by seeding in the pre-coated plates at 1 × 10^6^ cells/mL (1 mL/well). After 2 days, the cells were transduced with either Lenti/ECAR or Lenti/ECAR-EGFP viruses for a period of 24 h. The conventional ECAR-T cells were expanded for about 2 weeks in C10 medium and then cryopreserved for future applications.

### Generation of ^PBMC^ECAR-NKT cells

Healthy donor PBMCs were sorted with magnetic-activated cell sorting via Anti-iNKT Microbeads (Miltenyi Biotech) labeling to enrich NKT cells, following the manufacturer’s instructions. The enriched NKT cells were mixed with donor-matched irradiated αGC/PBMCs at a ratio of 1:1, followed by culturing in C10 medium supplemented with 10 ng/mL human IL-7 and IL-15. On day 3, NKT cells were transduced with Lenti/ECAR viruses for 24 h. The resulting ^PBMC^ECAR-NKT cells were expanded for about 2 weeks in C10 medium supplemented with 10 ng/mL human IL-7 and IL-15 and cryopreserved for future use.

### *In vitro* tumor cell killing assay

Human GBM tumor cells (i.e., U87MG-FG and U87MG-EGFRvIII-FG; 1 × 10^4^ cells per well in 96-well plate) were co-cultured with the indicated therapeutic cells (i.e., PBMC-T, ECAR-T, and ^Allo^ECAR-NKT cells) in Corning 96-well clear bottom black plates for 24 h in C10 medium. The effector-to-target cell ratio (E:T) is indicated in the figure legends. At the end of culture, viable tumor cells were quantified by adding D-luciferin (150 μg/mL; Fisher Scientific, catalog no. 50-209-8110) to cell cultures, followed by the measurement of luciferase activity using an Infinite M1000 microplate reader (Tecan). To test NK receptor-mediated tumor cell killing, 10 μg/mL Ultra-LEAF purified anti-human NKG2D (clone 1D11, BioLegend, catalog no. 320813) or anti-human DNAM-1 antibody (clone 11A8, BioLegend, catalog no. 338302) was added to co-cultures to investigate the tumor cell killing mechanism by ^Allo^ECAR-NKT cells, and LEAF purified mouse lgG2b κ isotype control antibody (clone MG2b-57, BioLegend, catalog no. 401202) was included as an isotype control.

### *In vitro* GBM/macrophage organoid targeting assay

Healthy donor PBMC-derived, M2-polarized macrophages were used in this assay. PBMCs were resuspended in serum-free RPMI 1640 medium (Corning Cellgro, catalog no. 10-040-CV) at 1 × 10^7^ cells/mL, plated in 10-cm dishes (10–15 mL per dish), and incubated at 37°C with 5% CO_2_ for 1 h. Non-adherent cells were removed, and adherent monocytes were washed twice with PBS and cultured in C10 medium supplemented with recombinant human M-CSF (10 ng/mL, PeproTech, catalog no. 300-25) for 6 days to generate macrophages. On day 6, the macrophages were detached using 0.25% trypsin/EDTA (GIBCO, catalog no. 25200-056), collected, and reseeded in 6- or 12-well plates (0.5–1 × 10^6^ cells/mL) for another 48 h with recombinant human IL-4 (10 ng/mL, PeproTech, catalog no. 214-14) and IL-13 (10 ng/mL, PeproTech, catalog no. 214-13) to induce polarization. M2-polarized macrophages were then harvested.

To generate tumor organoids, either 2 × 10^5^ U87MG tumor cells alone or a 1:1 mixture of 1 × 10^5^ U87MG tumor cells and 1 × 10^5^ M2 macrophages were resuspended in C10 medium at a concentration of 1 × 10^5^ cells/μL. Cell aggregates were prepared by dispensing 5–10 μL of the cell suspension onto microporous membrane inserts (EMD Millipore, catalog no. PICM0RG50) placed in 6-well plates containing 1 mL of C10 medium per well.^51^^,^^52^ After a 2-day incubation period to allow organoid formation, 1 × 10^6^ therapeutic cells (i.e., ECAR-T or ^Allo^ECAR-NKT cells) were resuspended in 100 μL C10 medium and added on top of each organoid. Co-culture was maintained for 24 h. Following the co-culture period, organoids were mechanically dissociated with a 1-mL pipette and passed through a 70-μm nylon strainer to generate single-cell suspensions for downstream flow cytometry analysis.

### *In vivo* antitumor efficacy study: U87MG-(EGFRvIII-)FG human GBM xenograft NSG mouse model

The experimental design is shown in Figures 4A and 4D. Briefly, on day 0, NSG mice received s.c. inoculation of human GBM tumor cells (U87MG-FG or U87MG-EGFRvIII-FG, 1 × 10^6^ cells per mouse). On day 5, the experimental mice received peritumor (p.t.) injection of vehicle (100 μL PBS per mouse), ^Allo^ECAR-NKT cells (2 × 10^6^ ECAR^+^ cells in 100 μL PBS per mouse), or control ECAR-T cells (2 × 10^6^ ECAR^+^ cells in 100 μL PBS per mouse). Over the experiment, mice were monitored for survival, and their tumor volumes were measured. Tumor volume was calculated using the formula V = (width^2^ × length)/2. At the terminal day, the experimental mice were euthanized, tumors were collected, and tumor weights were measured.

### *In vivo* dose-gradient comparison study: U87MG-EGFRvIII-FG human GBM xenograft NSG mouse model

The experimental design is shown in Figure S5A. Briefly, on day 0, NSG mice received s.c. inoculation of U87MG-EGFRvIII-FG cells (1 × 10^6^ cells per mouse). On day 5, the experimental mice received p.t. injection of vehicle (100 μL PBS per mouse), ^Allo^ECAR-NKT cells (0.5 × 10^6^, 1 × 10^6^, 2 × 10^6^, or 5 × 10^6^ ECAR^+^ cells in 100 μL PBS per mouse), or control ECAR-T cells (0.5 × 10^6^, 1 × 10^6^, 2 × 10^6^, or 5 × 10^6^ ECAR^+^ cells in 100 μL PBS per mouse). Over the experiment, the tumor volumes were measured. At the terminal day, the experimental mice were euthanized, tumors were collected, and tumor weights were measured.

### *In vivo* antitumor efficacy study: Patient-derived neurosphere GBM39 orthotopic xenograft NSG mouse model

The experimental design is shown in Figure 4L. Briefly, on day 0, 2 × 10^5^ GBM39 neurosphere cells were intracranially implanted into the forebrain of NSG mice to establish an orthotopic GBM model. Injection coordinates were 2 mm lateral and 1 mm posterior to bregma, at a depth of 2 mm. On day 30, 1 × 10^6^ ^Allo^ECAR-NKT cells or conventional ECAR-T cells were administered intratumorally. Throughout the study, mice were monitored for survival, and tumor burden was assessed via peripheral blood sampling and Gaussia luciferase analysis. To measure the levels of sGluc activity, 6 μL blood was collected from the tail vein and immediately mixed with 50 mM EDTA to prevent coagulation. sGluc activity was obtained by measuring chemiluminescence after mixture with 100 μL of 100 mM coelenterazine (Nanolight 303) in an opaque 96-well plate using a luminometer (BMG Labtech, Clariostar). Endpoints were determined primarily by body conditioning score, especially focusing on the 30% weight loss threshold, decreased mobility, uncontrollable seizures and/or bleeding, and respiratory distress. Other criteria under the animal research committee policy on humane treatment and endpoints was also assessed.

### *In vivo* safety study: Patient-derived neurosphere GBM39 orthotopic xenograft NSG mouse model

The experimental design is shown in Figures 4L and 5A. Body weight of the experimental mice was monitored regularly throughout the study. On day 45 (15 days post-cell injection), peripheral blood was collected via retro-orbital bleeding, and the presence of human T cells was assessed by flow cytometry using anti-human CD45 and CD3 antibodies. Serum samples were obtained for cytokine analysis, and levels of mouse IL-6 and SAA-3 were quantified using ELISA.

### *In vivo* CRS study

The experimental design is shown in Figure 5D. Briefly, on day 0, 5 × 10^6^ U87MG-EGFRvIII cells were injected i.p. into NSG mice to establish a high tumor burden, following previously established protocols.^63^ On day 10, mice received an i.p. injection of 1 × 10^7 Allo^ECAR-NKT or conventional ECAR-T cells. Body weight was monitored daily to assess treatment-related toxicity. On day 13, serum was collected for cytokine analysis, and mouse IL-6 and SAA-3 concentrations were quantified by ELISA.

### *In vivo* GvHD evaluation

The experimental design is shown in Figure S6C. Briefly, on day 0, NSG mice received s.c. inoculation of U87MG-EGFRvIII-FG cells (1 × 10^6^ cells per mouse). On day 5, the experimental mice received p.t. injection of vehicle (100 μL PBS per mouse), ^Allo^ECAR-NKT cells (2 × 10^6^ ECAR^+^ cells in 100 μL PBS per mouse), or control ECAR-T cells (2 × 10^6^ ECAR^+^ cells in 100 μL PBS per mouse). Over the experiment, mouse body weight and GvHD score were measured. A score ranging from 0 to 2 was assigned for each clinical GvHD sign, which includes body weight, activity, posture, skin thickening, diarrhea, and dishevelment.^34^

### Statistical analysis

GraphPad Prism 9 software was used for statistical data analysis. Student’s 2-tailed t test was used for pairwise comparisons. Ordinary 1-way ANOVA followed by Tukey’s or Dunnett’s multiple comparisons test was used for multiple comparisons. The log rank (Mantel-Cox) test adjusted for multiple comparisons was used for Meier survival curves analysis. Data are presented as the mean ± SEM, unless otherwise indicated. In all figures and figure legends, “*n*” represents the number of samples or animals used in the indicated experiments. A *p* < 0.05 was considered significant.

## Data availability

All data associated with this study are present in the paper or the supplemental information.

## Acknowledgments

We thank the 10.13039/100007185University of California, Los Angeles animal facility for providing animal support; the UCLA Translational Pathology Core Laboratory (TPCL) for providing histology support; the UCLA Technology Centre for Genomics & Bioinformatics (TCGB) facility for providing RNA sequencing services; the UCLA CFAR Virology Core for providing human cells; and the UCLA BSCRC Flow Cytometry Core Facility for cell sorting support. This work was supported by a Partnering Opportunity for Discovery Stage Research Projects Award and a Partnering Opportunity for Translational Research Projects Award from the 10.13039/100000900California Institute for Regenerative Medicine (DISC2-11157, DISC2-13015, TRAN1-12250, and TRAN1-16050 to L.Y.), a Department of Defense CDMRP PRCRP Impact Award (CA200456 to L.Y.), a Department of Defense Kidney Cancer Research Program Award (KC230215 to L.Y.), a UCLA BSCRC Innovation Award (to L.Y.), and an Ablon Scholars Award (to L.Y.). L.Y. is a member of the UCLA Parker Institute for Cancer Immunotherapy. Y.-R.L. is a postdoctoral fellow supported by a UCLA MIMG M. John Pickett Post-Doctoral Fellow Award, a CIRM-BSCRC Postdoctoral Fellowship, a UCLA Sydney Finegold Postdoctoral Award, a UCLA Chancellor’s Award for Postdoctoral Research, and a UCLA Goodman-Luskin Microbiome Center Collaborative Research Fellowship Award. Some figures were created with BioRender (biorender.com).

## Author contributions

Y.-R.L. designed the experiments, analyzed the data, and wrote the manuscript. L.Y. conceived and oversaw the study, with advice from D.A.N. and R.M.P. Y.-R.L. performed all experiments, with assistance from Y.Z., Z.L., X.S., T.H., C.T., Y.T., J.H., A.S.Z., N.Y.M., and C.Z.

## Declaration of interests

L.Y. is a scientific advisor to AlzChem and Amberstone Biosciences and a co-founder, stockholder, and advisory board member of Appia Bio. None of the declared companies contributed to or directed any of the research reported in this article.

## Supplemental information

Document S1. Figures S1–S6 and Table S1

Document S2. Article plus supplemental information
