# Supplementary material for: Allogeneic stem cell-engineered EGFRvIII-specific CAR-NKT cells for treating glioblastoma with enhanced efficacy and safety
Source: Mol Ther. 2025 Sep 12;33(12):6041–62. doi: 10.1016/j.ymthe.2025.09.026 (PMC12703166; doi:10.1016/j.ymthe.2025.09.026)
Supplement: Document S1. Figures S1–S6 and Table S1 [file mmc1.pdf]

YMTHE, Volume *33*

Supplemental Information

Allogeneic stem cell-engineered EGFRvIII-specific CAR-NKT cells for treating glioblastoma with enhanced efficacy and safety

Yan-Ruide Li, Yichen Zhu, Zhe Li, Xinyuan Shen, Tyler Halladay, Christopher Tse, Yanxin Tian, Jie Huang, Annabel S. Zhao, Nathan Y. Ma, Catherine Zhang, David A. Nathanson, Robert M. Prins, and Lili Yang

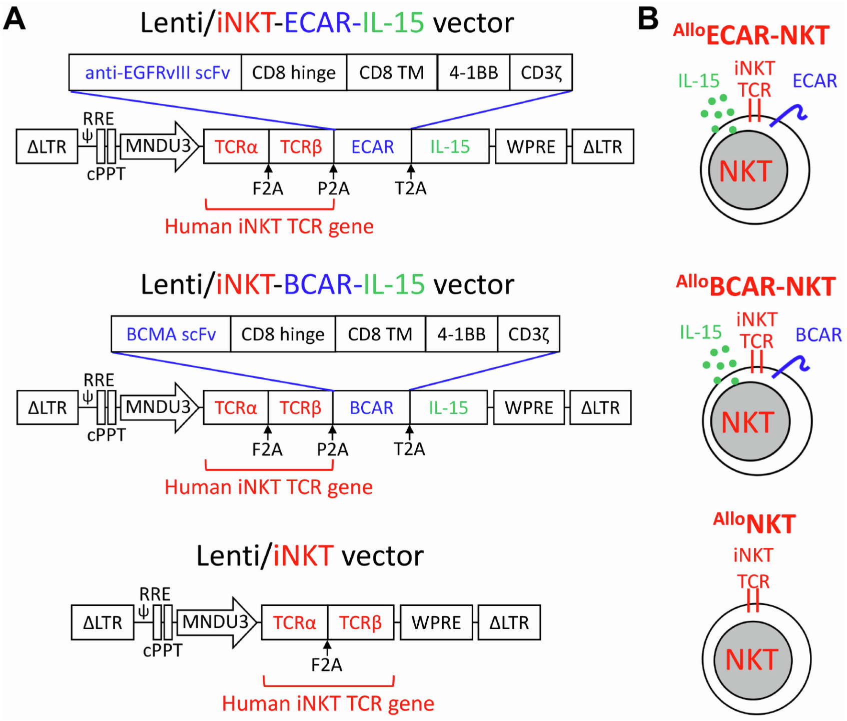

Figure S1. The lentivector designs (A) and allogeneic HSPC-derived NKT cells (B); related to Figure 1.

ΔLTR, self-inactivating long terminal repeats; MNDU3, internal promoter derived from the MND retroviral LTR U3 region; φ, packaging sequence; RRE, rev-responsive element; cPPT, central polypurine tract; WPRE, woodchuck hepatitis virus posttranscriptional regulatory element; F2A, foot-and-mouth disease virus 2 A; P2A, porcine teschovirus-1 2A; T2A, thosea asigna virus 2A; BCAR, BCMA-specific CAR.

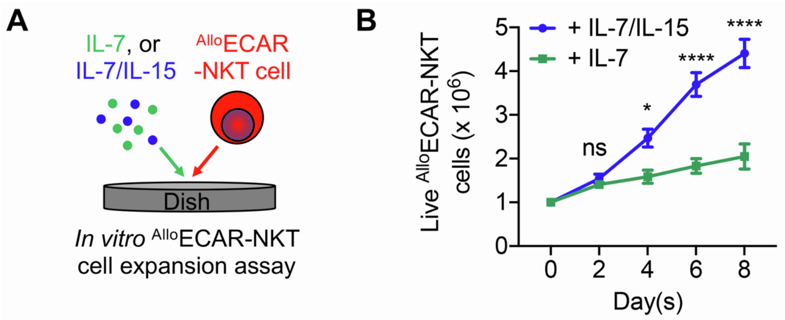

Figure S2. Evaluating cytokine requirements for AlloECAR-NKT cell expansion; related to Figure 1.

(A) Experimental design. AlloECAR-NKT cells were cultured *in vitro* under different cytokine conditions, including IL-7 alone and the combination of IL-15 and IL-7.

(B) Quantification of live AlloECAR-NKT cells over time ($n = 4$).

Representative of 3 experiments. Data are presented as the mean ± SEM. ns, not significant, *$p < 0.05$, ****$p < 0.0001$, by two-way ANOVA.

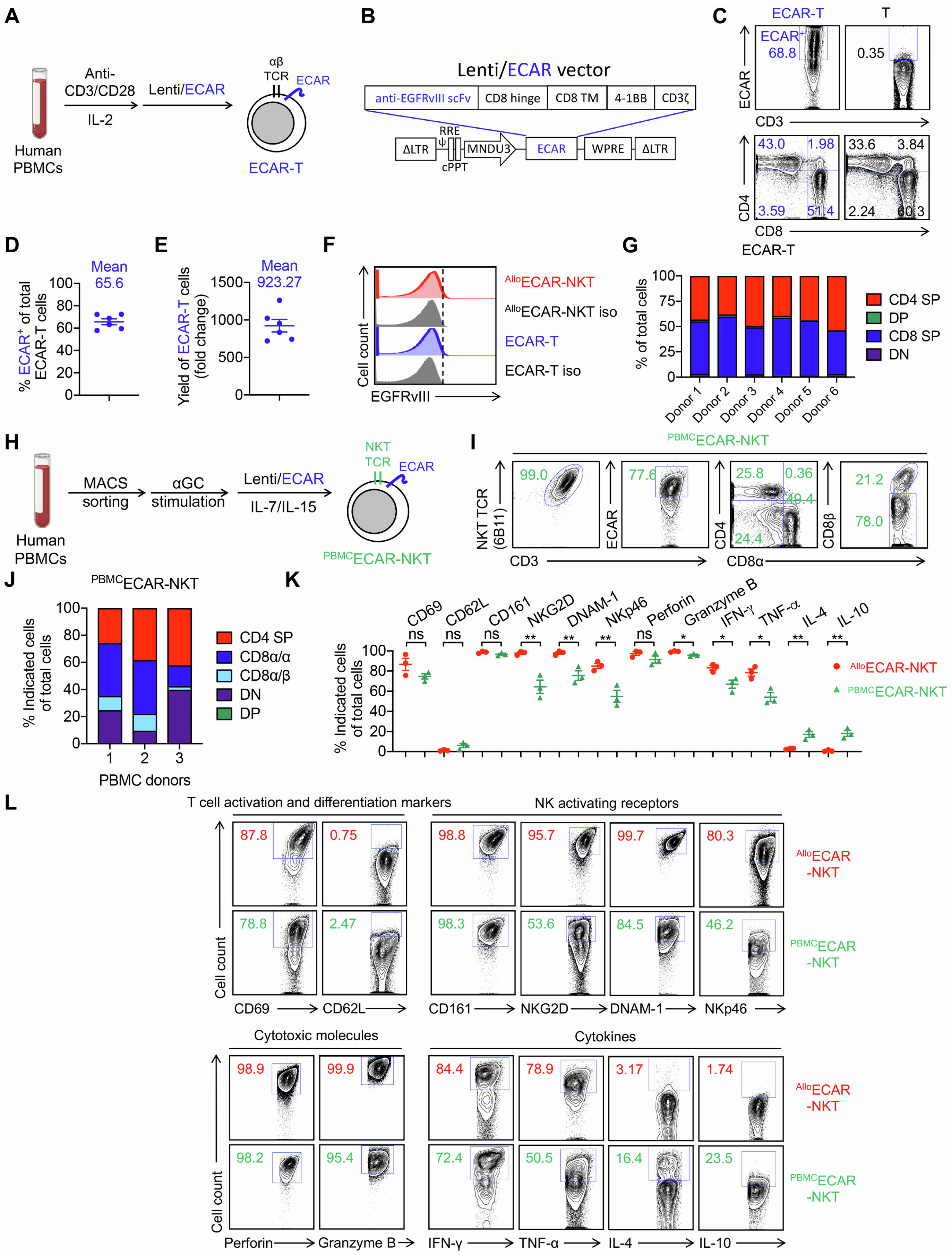
A
Human PBMCs
Anti-CD3/CD28
IL-2
Lenti/ECAR
αβ TCR
ECAR
ECAR-T
B
Lenti/ECAR vector
anti-EGFRvIII scFv
CD8 hinge
CD8 TM
4-1BB
CD3ζ
ΔLTR
RRE
ψ
cPPT
MNDU3
ECAR
WPRE
ΔLTR
C
ECAR-T
T
ECAR+ 68.8
0.35
ECAR
CD3
43.0
1.98
3.59
51.4
33.6
3.84
2.24
60.3
CD4
CD8
ECAR-T
D
Mean 65.6
% ECAR+ of total ECAR-T cells
E
Mean 923.27
Yield of ECAR-T cells (fold change)
F
AlloECAR-NKT
AlloECAR-NKT iso
ECAR-T
ECAR-T iso
Cell count
EGFRvIII
G
% of total cells
CD4 SP
DP
CD8 SP
DN
Donor 1
Donor 2
Donor 3
Donor 4
Donor 5
Donor 6
H
Human PBMCs
MACS sorting
αGC stimulation
Lenti/ECAR
IL-7/IL-15
NKT TCR
ECAR
PBMCECAR-NKT
I
PBMCECAR-NKT
99.0
77.6
25.8
0.36
49.4
24.4
21.2
78.0
NKT TCR (6B11)
ECAR
CD4
CD8β
CD3
CD8α
J
PBMCECAR-NKT
% Indicated cells of total cells
CD4 SP
CD8α/α
CD8α/β
DN
DP
PBMC donors
K
CD69
CD62L
CD161
NKG2D
DNAM-1
NKp46
Perforin
Granzyme B
IFN-γ
TNF-α
IL-4
IL-10
ns
ns
ns
**
**
**
ns
*
*
*
**
**
% Indicated cells of total cells
AlloECAR-NKT
PBMCECAR-NKT
L
T cell activation and differentiation markers
NK activating receptors
87.8
0.75
98.8
95.7
99.7
80.3
AlloECAR-NKT
78.8
2.47
98.3
53.6
84.5
46.2
PBMCECAR-NKT
Cell count
CD69
CD62L
CD161
NKG2D
DNAM-1
NKp46
Cytotoxic molecules
Cytokines
98.9
99.9
84.4
78.9
3.17
1.74
AlloECAR-NKT
98.2
95.4
72.4
50.5
16.4
23.5
PBMCECAR-NKT
Cell count
Perforin
Granzyme B
IFN-γ
TNF-α
IL-4
IL-10

Figure S3. Generation and characterization of conventional ECAR-T cells and PBMC-derived ECAR-NKT (^{PBMC}ECAR-NKT) cells; related to Figure 2.

(A-G) Generation and characterization of conventional ECAR-T cells. (A) Schematics showing the generation of conventional ECAR-T cells. (B) Schematics showing the design of Lenti/ECAR lentivector. (C) FACS detection of ECAR and CD4/CD8 expressions on ECAR-T cells. Non ECAR-engineered T cells were included as a control. (D) Percentage of ECAR$^+$ cells among total ECAR-T cells (n = 6; n indicates different healthy donors). (E) Yield of ECAR-T cells (n = 6). (F) FACS detection of EGFRvIII expression on ^{Allo}ECAR-NKT and conventional ECAR-T cells. (G) Comparison of CD4/CD8 subpopulation percentages of ECAR-T cells. Data generated from 6 different healthy donors were shown.

(H-L) Generation and characterization of ^{PBMC}ECAR-NKT cells. (H) Schematics showing the generation of ^{PBMC}ECAR-NKT cells. (I) FACS detection of NKT TCR, ECAR and CD4/CD8 expressions on ^{PBMC}ECAR-NKT cells. (J) Comparison of CD4/CD8 subpopulation percentages of ^{PBMC}ECAR-NKT cells. Data generated from 3 different healthy donors were shown. (K and L) FACS analyses of surface and intracellular marker expression in ^{Allo}ECAR-NKT and ^{PBMC}ECAR-NKT cells. Both quantification (K; n = 3, n indicates different PBMC donors) and FACS plot data (L) are presented.

Representative of over 6 (A-G) and 3 (H-L) experiments. Data are presented as the mean ± SEM. ns, not significant, *p < 0.05, **p < 0.01, by Student's *t* test (K).

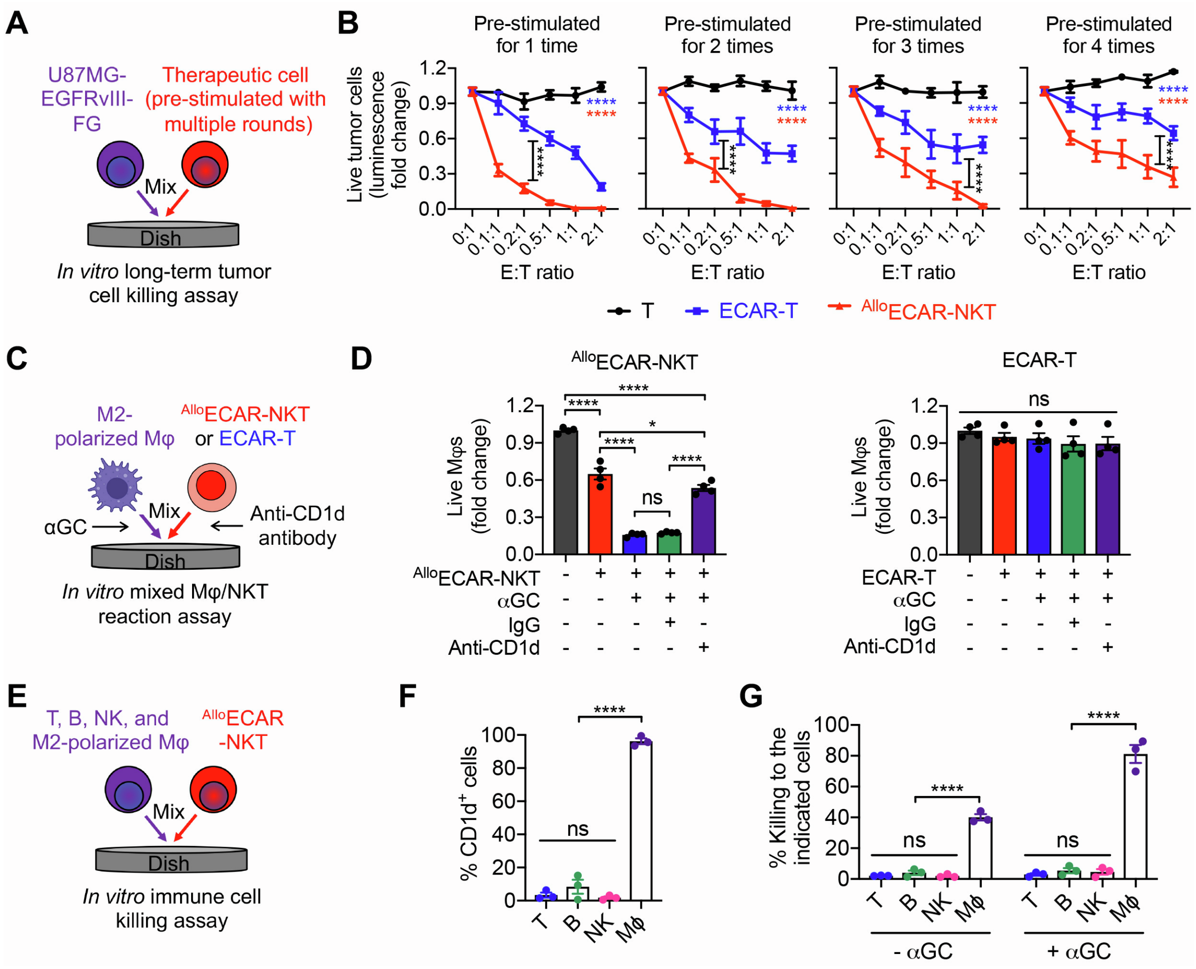

Figure S4. Studying the *in vitro* antitumor and anti-TME efficacy of AlloECAR-NKT cells; related to Figure 3.

(A and B) Studying the long-term *in vitro* antitumor efficacy of AlloECAR-NKT cells. (A) Experimental design. Therapeutic cells were subjected to multiple rounds (i.e., 1, 2, 3, 4, and 5 stimulations) of stimulation with U87MG-EGFRvIII-FG tumor cells, followed by collection and subsequent analysis. (B) Tumor cell killing data (n = 4).

(C and D) Studying AlloECAR-NKT cells targeting of GBM TME using *in vitro*-cultured human M2-polarized macrophages; related to Figures 3Q-3T. (C) Experimental design to study the direct killing to M2-polarized macrophages by AlloECAR-NKT and conventional ECAR-T cells. (D) M2-polarized macrophage killing data at 24 h (n = 4).

(E-G) Studying AlloECAR-NKT cells targeting of normal immune cells. (E) Experimental design. Healthy donor PBMC-derived T, B, and NK cells were tested, and human M2-polarized macrophages were included as a control. (F) FACS analyses of the percentage of $CD1d^+$ cells within the indicated immune cell populations (n = 3; n indicates different PBMC donors). (G) Immune cell killing data at 24 h (n = 4)

Representative of 3 experiments. Data are presented as the mean ± SEM. ns, not significant, *$p < 0.05$, ****$p < 0.0001$, by two-way ANOVA (B) and one-way ANOVA (D, F, and G).

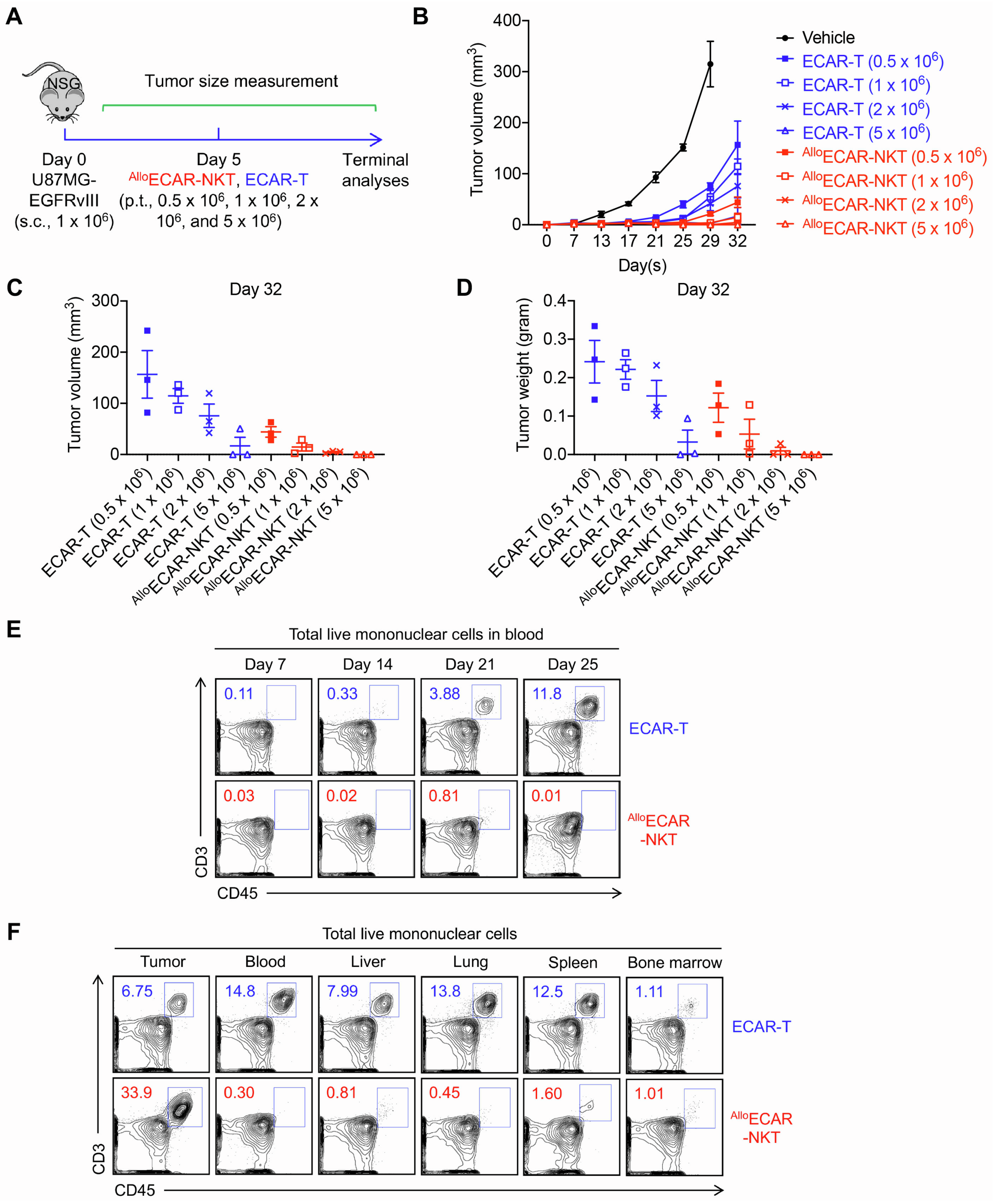

Figure S5. Studying the *in vivo* antitumor capacity of ^{Allo}ECAR-NKT cells; related to Figure 4.

(A-D) Dose-gradient comparison of ^{Allo}ECAR-NKT and ECAR-T cells in a U87MG-EGFRvIII human GBM xenograft mouse model. (A) Experimental design. (B) Tumor size measurements over time (n = 3). (C) Tumor size measurements on day 32 (n = 3). (D) Tumor weight measurements on day 32 (n = 3).

(E) FACS detection of ^{Allo}ECAR-NKT and ECAR-T cells in mouse peripheral blood over time; related to Figures 4G.

(F) FACS detection of ^{Allo}ECAR-NKT and ECAR-T cells in the indicated tissues at the terminal day (day 28); related to Figures 4H.
Data are presented as the mean ± SEM.

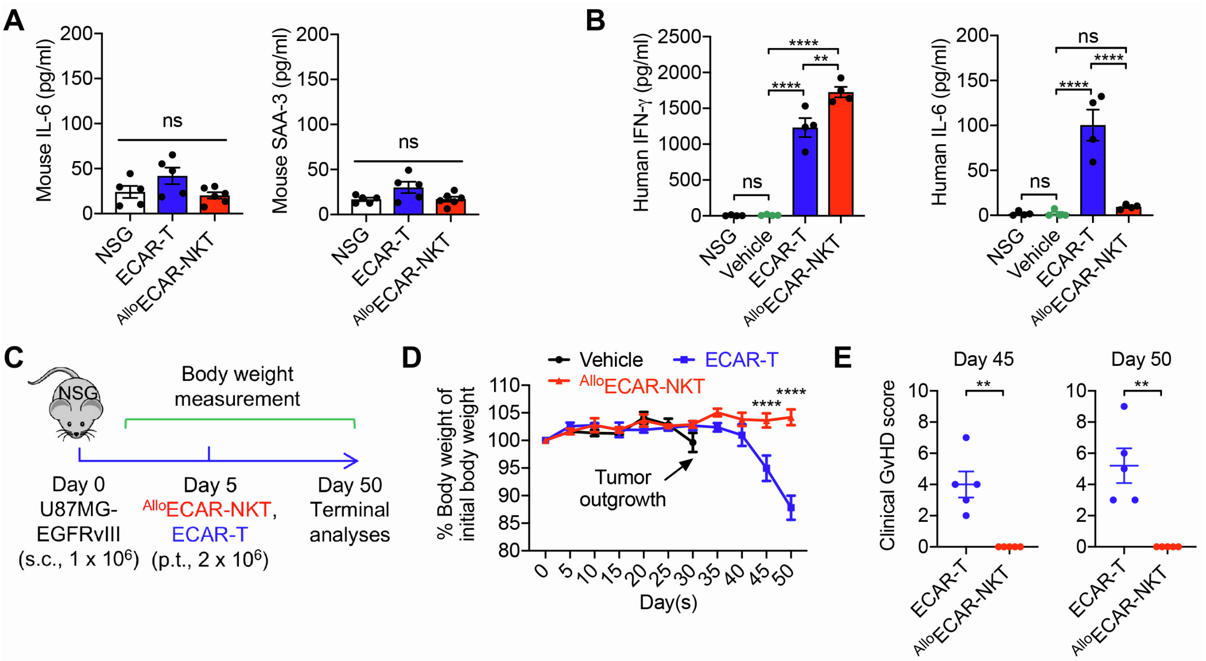

Figure S6. Studying the safety of AlloECAR-NKT cells; related to Figure 5.

(A) ELISA analyses of mouse IL-6 and SAA3 in mouse serum (n = 5-6); related to Figures 5A-5C.

(B) ELISA analyses of human IFN-γ and IL-6 in mouse serum (n = 4); related to Figures 5D-5F.

(C-E) Studying the graft-versus-host disease (GvHD) induced by AlloECAR-NKT and conventional ECAR-T cells; related to Figures 4A-4C. (C) Experimental design. (D) Body weight measured over time (n = 5). (E) Clinical GvHD score recorded over time (n = 5). The score was calculated as the sum of individual scores of 6 categories (body weight, activity, posture, skin thickening, diarrhea, and dishevelment; score 0-2 for each category).

Representative of 2 experiments. Data are presented as the mean ± SEM. ns, not significant, $^{**}p < 0.01$, $^{****}p < 0.0001$, by one-way ANOVA (A and B), two-way ANOVA (D), and Student's *t* test (E).

Table S1. Primary GBM patient sample information.

GBM lines	Diagnosis	Lobe	Sex
GBM39	Glioblastoma, IDH-wildtype, CNS WHO grade 4	Frontal	M
GS227	Glioblastoma, IDH-wildtype, CNS WHO grade 4	Right parietal	F
GS248	Glioblastoma, IDH-wildtype, CNS WHO grade 4	Right frontal	F

*IDH, Isocitrate Dehydrogenase; CNS WHO, Central Nervous System World Health Organization.
